# Synergistic p53 Pathway Activation Through Sono‐Gene Therapy Induced by Ultrasound‐Triggered Theranostic Mesoporous Nanoparticles

**DOI:** 10.1002/advs.76922

**Published:** 2026-08-03

**Authors:** Yading Zhao, Lu Guo, Dandan Shi, Xiao Sun, Mengmeng Shang, Song Ning, Shuting Huang, Xiaoxuan Wang, Rui Liu, Yuye Fu, Suyun Li, Jie Li

**Affiliations:** ^1^ Department of Ultrasound Qilu Hospital of Shandong University Jinan Shandong China

**Keywords:** CD24, p53, reactive oxygen species, sono‐gene therapy, ultrasound

## Abstract

Hepatocellular carcinoma (HCC) poses a significant global health burden due to its high mortality. The modest efficacy of single‐agent treatments is spurring the quest for combinatorial approaches. Sonodynamic therapy and gene therapy offer promising therapeutic approaches, yet they are limited by restricted tumor specificity and inefficient delivery. In this work, we developed a multifunctional mesoporous silica‐based nanoplatform co‐delivering indocyanine green (ICG) and CD24 small interfering RNA (siRNA) (siCD24), and the surface was engineered with lactobionic acid (LA) to enable active targeting and 2,3‐dimethylmaleic anhydride (DMMA) to confer tumor microenvironment‐responsive charge reversal, thereby enhancing specific accumulation and release. Under ultrasound irradiation, the nanoplatform concurrently activates dual mechanisms: ICG‐mediated sonodynamic action generates reactive oxygen species, while synchronized CD24 silencing modulates downstream effectors, such as p14 alternative reading frame and MDM2. This leads to coordinated regulation of the p53, resulting in the upregulation of the Cleaved caspase‐3 and p21, alongside the downregulation of Cyclin D1. Evaluations in vitro and in vivo demonstrated that combinatorial therapy exerts superior antitumor efficacy compared to monotherapy. Notably, the nanoparticles significantly enhanced ultrasound imaging. Our work validates the concept of a unified theranostic platform for sono‐gene therapy and ultrasound imaging, representing a promising advance in HCC treatment.

## Graphical abstract

Schematic representation of ultrasound‐mediated ICG/siCD24@MSN‐LCD from nanostructure to synergistic sono‐gene therapy. This nanoplatform targets ASGPR via the LCD shell, which dissociates to release loaded ICG and siCD24. The core mechanism involves ultrasound‐guided sonodynamic therapy by ICG and CD24 knockdown by siCD24, both activating the p53 axis to inhibit tumor progression. Combined with ultrasound imaging, it achieves integrated HCC theranostics.

## 1 Introduction

The clinical profile of hepatocellular carcinoma (HCC) is marked by insidious onset, aggressive progression, and a typically grave prognosis [1, 2]. Despite recent advances in diagnostic and therapeutic technologies, HCC continues to pose a significant challenge due to its poor overall prognosis and alarmingly low 5‐year survival rate, particularly in late‐stage disease [3, 4], underscoring the urgent need for innovative treatment strategies. Moreover, the disconnect between diagnosis and treatment can exacerbate tissue damage and side effects, which also limits the overall efficiency and precision of anticancer therapy. Therefore, the development of an integrated theranostic platform that combines diagnostic and therapeutic functions holds significant potential for improving cure rates and survival outcomes in HCC patients.

CD24 is well‐established as both an overexpressed oncoprotein and a poor prognostic marker in various malignancies, such as oesophageal squamous cell carcinoma [5], HCC [6], breast cancer, and ovarian cancer [7]. Studies employing antibody blockade, gene silencing, and ectopic or inducible expression have confirmed its contribution to tumor growth [8, 9]. Beyond proliferation, CD24 also participates in metastasis [10, 11]. The underlying mechanism involves fucosylated CD24 binding to P‐ and E‐selectins, which mediates endothelial rolling and promotes distant invasion [12]. Interestingly, a groundbreaking study in prostate cancer reveals that p14 alternative reading frame (ARF) is stabilized within the nucleolus through its interaction with nucleophosmin (NPM), while CD24 competitively inhibits the binding between ARF and NPM. This leads to the destabilization of ARF, resulting in increased MDM2 abundance and ultimately a reduction in p53 [13]. This finding positions CD24 as a novel upstream oncogenic regulator. We hypothesize that a similar CD24‐ARF‐p53 regulatory axis may exist in HCC, and silencing CD24 expression could represent a promising therapeutic strategy.

However, achieving efficient gene therapy faces multiple challenges, including the inherent instability of nucleic acid drugs in vivo and their insufficient targeting capability [14, 15]. In recent years, nanomedicine has emerged as a promising strategy for gene/drug delivery and enhanced cancer therapy. Among various nanocarriers, mesoporous silica nanoparticle (MSN) has attracted significant interest due to their unique advantageous properties [16, 17], such as robust mesoporous frameworks, high surface area, tunable pore dimensions, and volumes, as well as modifiable surface characteristics [18]. Moreover, recent review articles have well documented that MSN serves as excellent nanocarriers for small interfering RNA (siRNA) delivery [19, 20, 21], thereby enabling efficient CD24 gene silencing. Despite the promising potential of MSN for gene therapy, conventional MSN‐based platforms still suffer from several typical drawbacks [22, 23], including insufficient active targeting ability, lack of tumor microenvironment‐responsive release behavior, and single therapeutic modality. To address the limitation of single therapeutic modality and further enhance antitumor efficacy via synergistic therapy, we loaded a sonosensitizer into MSN to realize combined gene therapy and sonodynamic therapy (SDT) under ultrasound (US). Furthermore, the MSN‐based system demonstrates favorable US imaging performance, laying a foundation for the development of image‐guided synergistic theranostic platforms [24].

Apart from the above improvement of therapeutic modality, the insufficient active targeting capability and lack of tumor microenvironment‐responsive release still remain to be optimized. Extensive studies have demonstrated that rational framework reconstruction, surface functionalization, and morphology design can effectively address these limitations and endow MSN with improved responsiveness and delivery performance [25, 26, 27]. Unlike conventional single‐component modification, we employed biocompatible natural chitosan (CS) as the coating layer [28], whose abundant hydroxyl and amino groups provide sufficient reactive sites for subsequent dual‐functional decoration with lactobionic acid (LA) and 2,3‐dimethylmaleic anhydride (DMMA). Specifically, LA conjugation enables the nanoplatform to actively target hepatocellular carcinoma cells (e.g., Huh7) by specifically recognizing the asialoglycoprotein receptor (ASGPR) [29]. Meanwhile, DMMA forms acid‐labile β‐carboxylic amide bonds with chitosan, endowing the platform with pH‐triggered charge reversal responsiveness within the tumor microenvironment [30]. This dual‐functional design achieves precise tumor targeting and site‐specific drug/gene release, effectively improving therapeutic efficacy, and reducing non‐specific off‐target damage.

**SCHEME 1:**
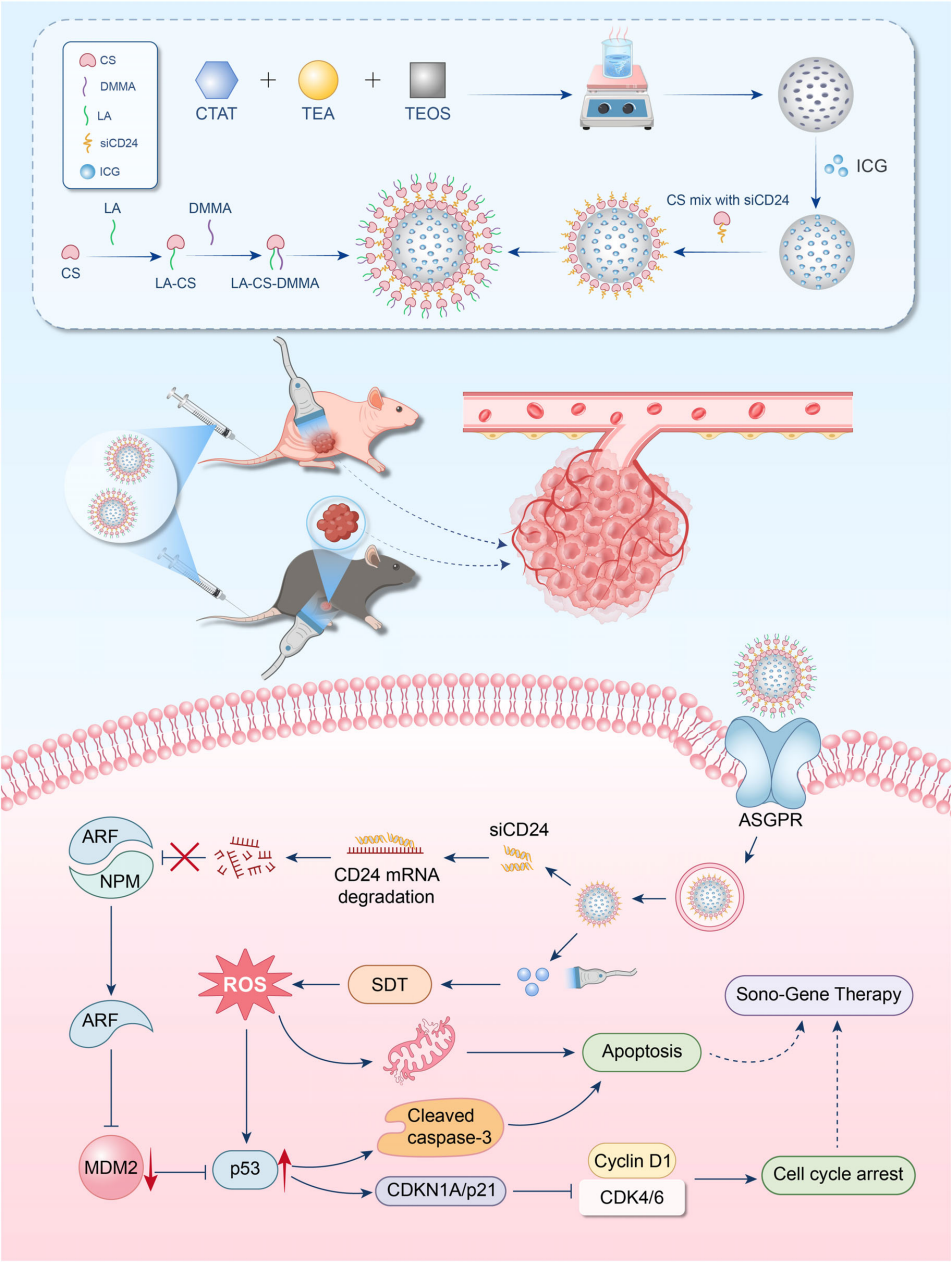
Schematic representation of ultrasound‐mediated ICG/siCD24@MSN‐LCD from nanostructure to synergistic sono‐gene therapy in HCC. ICG/siCD24@MSN‐LCD actively targets asialoglycoprotein receptor (ASGPR) via its LA‐CS‐DMMA (LCD) shell, and the LCD coating dissociates and sheds off specifically after binding, followed by the release of loaded ICG and siCD24, which exert synergistic effects. The core mechanism involves ultrasound‐guided sonodynamic therapy (SDT) by ICG and CD24 knockdown by siCD24, both activating the p53 axis to inhibit tumor progression. Specifically, on the one hand, siCD24 mediates CD24 knockdown to release the competitive inhibition of ARF‐nucleophosmin (NPM) interaction, the restored ARF stability suppresses MDM2 activity, thereby attenuating MDM2‐induced p53 ubiquitination and degradation. On the other hand, ICG serves as a sonosensitizer to generate ROS under ultrasound, and the produced ROS also participates in activating the p53 signaling pathway. The accumulated p53 further activates downstream target genes including p21/CDKN1A to realize synergistic sono‐gene therapy. Integrated with ultrasound imaging capabilities, it constitutes a novel all‐in‐one theranostic strategy for HCC.

Encouraged by the above background and structural design advantages, we herein fabricated a novel dual‐functional modified MSN nanoplatform loaded with indocyanine green (ICG) and CD24 siRNA (siCD24), and coated with an LA‐CS‐DMMA (LCD) outer protective shell, which integrates targeted responsiveness, synergistic sono‐gene therapy, and US imaging capabilities (Scheme 1). When compared with reported similar nanotherapeutic systems, polythiophene‐based platforms have achieved SDT‐gene synergistic therapy [31], yet they are constructed with soft organic frameworks rather than rigid porous MSN structure, and fail to integrate imaging guidance as well as tumor microenvironment‐responsive charge reversal. Moreover, most current MSN‐based sonodynamic platforms merely combine SDT with chemotherapy, hyperthermia, or immunotherapy [32, 33, 34], while few designs introduce specific gene silencing to intervene key oncogenic signaling pathways. Distinct from these reported designs, our nanoplatform simultaneously integrates tumor targeting, microenvironment‐triggered charge reversal, co‐delivery of sonosensitizer and therapeutic gene, and US imaging‐guided therapy, which is rarely reported in conventional MSN designs. Notably, almost no MSN‐based nanoplatform has been engineered to regulate the CD24‐ARF‐MDM2‐p53 signaling axis for HCC therapy, which further highlights the novelty and originality of our design. Herein, we systematically characterize the physicochemical properties, in vitro performance and in vivo therapeutic efficacy of the as‐prepared nanoplatform. This multifunctional nanoplatform is expected to efficiently target HCC and suppress tumor growth via synergistic sono‐gene therapy, offering a promising experimental foundation for targeted clinical HCC therapy.

## 2 Experimental Section

### 2.1 Materials

Hexadecyltrimethylammonium p‐toluenesulfonate (CTAT), tetraethyl orthosilicate (TEOS), and triethylamine (TEA) are obtained by Sigma‐Aldrich (MO, USA). Chitosan, RNase A solution, and crystal violet are sourced from Solarbio (Beijing, China). LA is procured from Bide (Shanghai, China). DMMA and ICG are acquired from Macklin (Shanghai, China). Human/Mouse CD24 siRNA and EdU‐567 Kit are supplied by RiboBio (Guangzhou, China). The sequence of human CD24 siRNA was CAACTGGAACTTCAAGTAA, and the sequence of mouse CD24 siRNA was CCCGGTAACCAGAATATTT. 2′,7′‐Dichlorodihydrofluorescein diacetate (DCFH‐DA), Dihydroethidium (DHE), and the Live/Dead Staining Kit are supplied by Beyotime (Shanghai, China). Rabbit anti‐CD24 is purchased from Abcam (Cambridge, UK). Rabbit anti‐ARF, rabbit anti‐p53, rabbit anti‐p21, and Cleaved caspase‐3 antibodies are purchased from Cell Signaling Technology (Boston, USA). Mouse anti‐MDM2 antibody is obtained from Santa Cruz (California, USA). Rabbit anti‐Cyclin D1 antibody is from Abways (Shanghai, China). Rabbit anti‐Ki67 antibody and TUNEL kit are procured by Servicebio (Wuhan, China). Hematoxylin and eosin (HE) staining kit is provided by Ketu (Hefei, China). 0.25% trypsin‐EDTA, Dulbecco's Modified Eagle Medium (DMEM), and fetal bovine serum (FBS) are purchased from Gibco (USA).

### 2.2 Synthesis of LCD

LA (200 mg), 1‐Ethyl‐3‐(3‐dimethylaminopropyl) carbodiimide hydrochloride (EDC) (214 mg), and *N*‐Hydroxysuccinimide (NHS) (128 mg) were dissolved in pure water and stirred at room temperature for 2 h. CS (1.4 g) was then added, and the reaction proceeded over 48 h under ambient conditions after adjusting the pH to 5 with NaOH. The resulting mixture was precipitated in anhydrous ethanol, washed twice, and dried with ice‐cold ether to obtain the product LA‐CS. Subsequently, a solution of LA‐CS (500 mg) in DMSO (15 mL) was prepared, to which was added 150 mg of DMMA and TEA (131 µL). Following a 24 h reaction, the compound was subjected to precipitation in anhydrous ethanol, washing twice with acetone, and drying with ice‐cold ether to yield the target compound LCD. LCD was assessed by ^1^H NMR spectrum (Qone AS 400, Zhongke‐Niujin, Wuhan, China) and FTIR (ZRAffinity‐1S, SHIMADZU, Tokyo, Japan).

### 2.3 Synthesis of MSN and ICG/siCD24@MSN‐LCD

MSN was fabricated according to a reported procedure [35]. First, a mixture of CTAT (960 mg) and TEA (174 mg) in water (50 mL) underwent magnetic stirring at 80°C. After the drop by drop addition of TEOS (7300 mg), the mixture was maintained for 2 h. Calcination at 550°C was performed on resultant nanoparticles following three washes using water/ethanol. Subsequently, the precipitate was redispersed in deionized water, and ICG solution was mixed with MSN at a mass ratio of 1:5, and stirred continuously in the dark for 8 h to obtain ICG@MSN. CS (0.4 mg) and siCD24 (26.4 µg) were mixed and incubated for 30 min, with subsequent incubation with ICG@MSN (9 mg, calculated based on the initial feeding mass of MSN) for 1 h. The complexes were then centrifuged, resuspended, and incubated overnight at 4°C with the pre‐synthesized LCD or Rhodamine B‐labeled LCD (Rhodamine B‐LCD), where the mass ratio of initial MSN to LCD or Rhodamine B‐LCD was 5:1. Finally, the products, denoted as ICG/siCD24@MSN‐LCD or Rhodamine B‐labeled ICG/siCD24@MSN‐LCD, were collected after centrifugation with RNase‐free water.

### 2.4 Immunohistochemical Analysis of CD24 in HCC

Immunohistochemical staining for CD24 was conducted on formalin‐fixed, paraffin‐embedded sections of human HCC, and paired adjacent non‐tumorous liver tissues. Tissue sections were deparaffinized, rehydrated, and subjected to antigen retrieval under heating conditions. After blocking endogenous peroxidase activity and non‐specific binding sites, tissues received successive incubations with CD24 primary antibody at 4°C overnight and horseradish peroxidase (HRP)‐conjugated secondary antibody. Finally, immunostaining was visualized by DAB development, and all tissue sections were counterstained with hematoxylin for morphological observation.

### 2.5 Characterization of ICG/siCD24@MSN‐LCD

Size, polydispersity index (PDI), and zeta potential were detected using Zeta potential and particle size analyzer (Malvern, UK). The morphology of MSN and ICG/siCD24@MSN‐LCD was identified utilizing transmission electron microscopy (TEM, JEOL, Japan) and scanning electron microscope (SEM, ZEISS, Germany). Elemental composition and chemical states of the incorporated components were determined using energy dispersive spectrometer (EDS) and X‐ray photoelectron spectroscopy (XPS). Brunauer–Emmett–Teller (BET) surface area and pore size were measured by an ASAP 2460 analyzer (Micromeritics, England). XRD patterns (SmartLab SE, Rigaku, Japan) were acquired using a powder diffractometer for X‐rays in the range of 10°–80° (2θ).

### 2.6 Encapsulation and Release of ICG/siCD24@MSN‐LCD

To assess the pH‐induced charge reversal of ICG/siCD24@MSN‐LCD, the nanoparticles were dispersed into phosphate buffered saline (PBS) at pH 7.4 or 6.5, respectively, then the zeta potential was measured. ICG content in ICG/siCD24@MSN‐LCD was determined by a UV–vis spectrometer at 780 nm (UV‐2600, Japan). The siCD24 content in ICG/siCD24@MSN‐LCD was quantified by a fluorescence microplate reader (Infinite E plex, Tecan, Switzerland), where siCD24 was labeled with FAM. For the in vitro release study, ICG/siCD24@MSN‐LCD was dispersed in PBS at different pH values (pH 7.4/6.5) in a shaker at 37°C. At predetermined time intervals, samples were collected and analyzed to determine the cumulative release profiles. To test the RNA protective effect of ICG/siCD24@MSN‐LCD, naked siRNA and ICG/siCD24@MSN‐LCD were incubated with RNase A for 1 and 24 h, separately. The subsequent integrity of the RNA was examined by agarose gel electrophoresis.

### 2.7 Cell Culture

The human HCC cell line (Huh7) and the mouse HCC cell line (Hepa1‐6) were maintained with DMEM containing 10% FBS, penicillin (100 U/mL), and streptomycin (100 mg/mL). The cells were cultured in a humidified incubator containing 5% CO_2_ at 37°C. US irradiation parameters were 1.0 W/cm^2^, 30 s, 1.0 MHz. All experiments were carried out with strict light avoidance.

### 2.8 In Vitro Gene Silencing Efficiency

Huh7 cells were allowed to adhere overnight prior to treatment. Two groups of cells were transfected with 50 nM siCD24 and negative control siRNA (siNC), respectively, via jetPRIME transfection reagent in strict accordance with the manufacturer's standard protocol. Meanwhile, another group of cells was treated with ICG/siCD24@MSN‐LCD at a concentration of 112.5 µg/mL (calculated as MSN equivalent), followed by US stimulation (1.0 MHz, 1 W/cm^2^, 30 s). All cell samples were continuously cultured for another 36 h after the above treatments. Subsequently, proteins were extracted, and the protein expression levels were detected via western blot. Relative protein expression levels were quantified by Image J.

### 2.9 Biocompatibility of MSN

The biocompatibility in vitro of the MSN was evaluated by live–dead cell staining. Huh7 cells were seeded into 96‐well culture plates and incubated overnight for attachment, then exposed to MSN suspensions at gradient concentrations for 24 h of continuous incubation. After rinsing with PBS, cells were co‐stained with Calcein‐AM and Propidium Iodide (PI) working solution for 30 min at 37°C in the dark. The cell viability was subsequently observed and recorded under a fluorescence microscope.

To assess the hemocompatibility of MSN, a hemolysis assay was conducted. The erythrocytes were subjected to centrifugation after being incubated with a series of MSN concentrations for 1 h at 37°C. A microplate reader was used to quantify the absorbance at 545 nm. Positive and negative controls were established using erythrocytes treated with ultrapure water and 0.9% NaCl solution, correspondingly. The percentage of hemolysis was then determined using the formula: Hemolytic rate(%) = (ODsample‐ODnegative)/(ODpositive‐ODnegative)×100%.

Mice were intravenously injected with 100 µL of MSN suspension (2.5 mg/mL) via the tail vein, while the control group received an equal volume of sterile normal saline under the same conditions. After 1 week, the mice were euthanized, and blood samples were collected for biochemical parameters (including alanine aminotransferase (ALT), aspartate aminotransferase (AST), blood urea nitrogen (BUN), and creatinine (CREA)).

### 2.10 US Imaging

Recently, evidence suggests that silica‐based nanoparticles enable US imaging. Just as there is an impedance mismatch at the interface between tissue and gaseous microbubbles, there is also an impedance mismatch at the interface between tissue and rigid silica particles [36, 37]. Building upon this principle, the present work aimed to investigate the US imaging potential of our silica‐based nanoparticles. US imaging was conducted using a US system fitted with a 9L linear transducer (GE LOGIQ E9, USA). For in vitro US imaging, a latex glove finger model containing the ICG/siCD24@MSN‐LCD solutions (2.5 mg/mL, calculated as MSN equivalent) at various concentrations was submerged in degassed water. The imaging parameters included a center frequency of 9.0 MHz, mechanical index (MI) of 1.0/0.5/0.26, dynamic range of 60 dB. For in vivo US imaging, the tumor‐bearing mice were injected with 100 µL of ICG/siCD24@MSN‐LCD (2.5 mg/mL, calculated as MSN equivalent) via tail vein. The major parameters are: 9 L linear transducer; center frequency of 9.0 MHz; MI of 1.0/0.5; dynamic range of 60 dB. PBS injection group served as the control.

### 2.11 Cellular Adhesion

Initial observation of cellular adhesion for Rhodamine B‐labeled ICG/siCD24@MSN‐LCD was performed using confocal laser scanning microscopy (CLSM). Huh7 cells were incubated with the nanoparticles at a concentration of 112.5 µg/mL (calculated as MSN equivalent) for 1, 4, 10, and 24 h. The LA‐treated/untreated cells were both incubated with nanoparticles for 10 h, with LA‐pretreated cells receiving a 2 h incubation with LA. Cells were counterstained with Hoechst 33 342 for nuclei. Imaging was then performed using a CLSM system (Olympus SpinSR10, Japan). To further quantify the cellular adhesion of the nanoparticles, flow cytometry (FCM) was conducted. After consistent treatment as described above, cells were harvested, washed, resuspended to form a single‐cell suspension, and then assessed by flow cytometer (Beckman Cytoflex, China). Meanwhile, the binding of ICG/siCD24@MSN‐LCD to Huh7 cells was observed using field emission scanning electron microscopy.

### 2.12 In Vivo Fluorescence Imaging and Biodistribution

Huh7 tumor‐bearing BALB/c nude mice were injected via tail vein with 100 µL of Rhodamine B‐labeled ICG/siCD24@MSN‐LCD (2.5 mg/mL, calculated as MSN equivalent). The mice were subjected to gaseous anesthesia and imaged at predetermined time points (1, 2, 3, 6, 9, and 24 h) post‐injection by an IVIS Spectrum system (PerkinElmer, USA). To further investigate the in vivo biodistribution of nanoparticles, mice were sacrificed at 2 and 24 h post‐injection. Tumor tissues and major organs were harvested and subjected to IVIS imaging. Subsequently, tumor tissues were harvested for cryosectioning, followed by DAPI staining, and intra‐tumoral fluorescence distribution was conducted by fluorescence microscopy.

### 2.13 Detection of Reactive Oxygen Species (ROS) and Superoxide Anion

Intracellular ROS generation was monitored using the fluorescent probe DCFH‐DA. The fluorogenic probe DHE is commonly used for detection of superoxide anion generation in cells. Huh7 cells were divided into six groups. G1: Control; G2: US; G3: ICG@MSN‐LCD+US; G4: siCD24@MSN‐LCD+US; G5: ICG/siCD24@MSN‐LCD; G6: ICG/siCD24@MSN‐LCD+US. The final concentrations of ICG and siCD24 were 16.4 µg/mL and 24.3 nM, respectively. In the US‐related groups, cells were subjected to US stimulation at 1 W/cm^2^ for 30 s. After 24 h of treatment, cells were incubated with DCFH‐DA/DHE in the dark for 20 min at 37°C. ROS/superoxide anion were, respectively, analyzed by inverted fluorescence microscopy.

### 2.14 Mitochondrial Membrane Potential Assay

Mitochondrial membrane potential was measured using a JC‐1 staining kit. Following the same grouping and treatment conditions as described above, Huh7 cells were stained using the JC‐1 in the dark for 30 min at 37°C. At high mitochondrial membrane potential, JC‐1 forms aggregates that emit red fluorescence, whereas at low membrane potential, it remains in monomeric form, producing green fluorescence. Finally, changes in JC‐1 fluorescence were analyzed using fluorescence microscopy.

### 2.15 Western Blotting

After identical grouping and 24 h treatment as described above, total cellular proteins were extracted from Huh7 cells. Protein samples were separated by SDS‐PAGE gels and electrotransferred to 0.22 µm PVDF using a Bio‐Rad system. Proteins were blocked for 1 h at room temperature before being incubated with primary antibodies against CD24, ARF, MDM2, p53, and p21 overnight at 4 °C. Following three washes with TBST, the membranes were probed with corresponding secondary antibodies at room temperature for 1 h. Protein bands were imaged using an ECL reagent and their relative levels were quantified with Image J software.

### 2.16 Cell Immunofluorescence

Following the same grouping and 24 h treatment conditions as described above, Huh7 cells cultured on coverslips were rinsed with PBS, fixed in 4% paraformaldehyde at room temperature, permeabilized with 0.1% Triton X‐100, and then blocked with 5% bovine serum albumin (BSA) to eliminate non‐specific antibody binding. Prior to immunofluorescence, overnight incubation with primary antibodies against CD24, p53, p21, Cyclin D1, and Cleaved caspase‐3 was conducted at 4°C, followed by a 1‐h incubation with corresponding fluorescent secondary antibodies. Nuclei were then labeled by DAPI, and the fluorescence was visualized under a CLSM.

### 2.17 RNA Sequencing

Huh7 cells were seeded into 6‐well culture plates and incubated overnight to allow complete adherence. The cells were then divided into the control group and the ICG/siCD24@MSN‐LCD combined with US (1 W/cm^2^ for 30 s) stimulation group, and continuously treated for 36 h. The final concentrations of ICG and siCD24 were 16.4 µg/mL and 24.3 nM, respectively. After treatment, cells were collected and lysed to extract total RNA. The qualified RNA samples were used for library construction with AT4221‐U‐mRNAseq Library Prep Kit (KAITAI‐Bio, AT4221), and subsequently subjected to transcriptome sequencing using the Illumina NovaSeq 6000 platform.

### 2.18 In Vitro Therapeutic Effect

Cell proliferation is assessed through EdU kit following manufacturer's protocol. Huh7 cells were exposed to six treatments. G1: Control; G2: US; G3: ICG@MSN‐LCD+US; G4: siCD24@MSN‐LCD+US; G5: ICG/siCD24@MSN‐LCD; G6: ICG/siCD24@MSN‐LCD+US. The final concentrations of ICG and siCD24 were 16.4 µg/mL and 24.3 nM, respectively. In the US‐related groups, cells were subjected to US stimulation at 1 W/cm^2^ for 30 s. After 24 h of treatment, Huh7 cells were incubated with EdU working solution at 37°C in a humidified 5% CO_2_ incubator for 2 h and then subjected to fixation, permeabilization, and Apollo fluorescent staining. Subsequently, cell nuclei were counterstained, and the fluorescence images were captured using an inverted fluorescence microscope for quantitative analysis of cell proliferation.

Cell apoptosis was evaluated using TUNEL staining. After identical treatment and grouping as aforementioned for 24 h, cells were gently washed with PBS, followed by sequential fixation, permeabilization, and TUNEL staining operations, which were all carried out strictly in compliance with the manufacturer's standard protocols. Finally, apoptotic fluorescence signals were observed and photographed under an inverted fluorescence microscope, and the apoptotic rate was analyzed based on the fluorescence images.

The migratory and invasive capabilities of Huh7 cells are examined using Transwell assays. Cells were seeded in 6‐well plates and subjected to the same treatments as mentioned above for 24 h. Subsequently, the cells were trypsinized, collected, and resuspended in serum‐free DMEM medium. 1 × 10^5^ treated cells were seeded into the upper chamber, which was un‐layered (for migration)/pre‐layered by Matrigel (for invasion). The bottom chambers of the Transwell plates were filled with medium containing 20% FBS. After incubation for 24 h, the non‐migrated/non‐invaded cells on the upper surface of the membrane were gently wiped off with a cotton swab. The migrated and invaded cells adhering to the lower surface of the membrane were fixed, stained with crystal violet, and then rinsed with PBS. Finally, the cells were photographed under an optical microscope, and the number of migrated/invaded cells was counted for quantitative evaluation.

Viability is evaluated by Live/Dead Staining. After receiving uniform experimental interventions as previously described for 24 h, Huh7 cells were gently rinsed with PBS to remove residual culture medium. Subsequently, the cells were co‐incubated with Calcein‐AM and PI working solution at 37°C for 30 min in the dark. Upon completion of staining, the fluorescence signals of live and dead cells were observed and photographed under a fluorescence microscope for intuitive assessment of cell survival status.

### 2.19 Animals

Male BALB/c nude mice (4–6 weeks old) were purchased from Beijing HFK Bio‐Technology Co., Ltd. Male C57BL/6 mice (4–6 weeks old) were obtained from Beijing Vital River Laboratory Animal Technology Co., Ltd. They were kept in specific pathogen‐free (SPF) conditions in the Animal Center of Qilu Hospital, Shandong University. All animal experiments were approved by the Animal Care and Use Committee of Qilu Hospital of Shandong University (DWLL‐2023‐106).

### 2.20 Establishment of a Hepatocellular Carcinoma Xenograft Model and Treatment

For tumor induction in nude mice, 1 × 10^7^ Huh7 cells resuspended in PBS were inoculated subcutaneously in the right flank. Mice were further treated once tumor volumes reached ∼50 mm^3^. Tumor‐bearing mice were randomly assigned to six groups (*n* = 5) as follows: Control (G1), US (G2), ICG@MSN‐LCD + US (G3), siCD24@MSN‐LCD + US (G4), ICG/siCD24@MSN‐LCD (G5), and ICG/siCD24@MSN‐LCD + US (G6) (ICG dosage: 2 mg/kg, siCD24 dosage: 40 µg/kg). All formulations were administered via tail vein injection at a volume of 100 µL. Mice in the control group received an equal volume of saline through tail vein injection. In the US related‐treated groups, tumors were subjected to US stimulation at 1.5 W/cm^2^ for 2 min post‐injection. All mice were treated on an every‐3‐day schedule (Day 0, 3, 6, and 9). Tumor volume and body weight are recorded every 2 days by the digital caliper and an electronic balance using an equation: V = L×W^2^/2, where L and W represent the longest and shortest diameters, respectively. At the treatment endpoint on day 14, they are euthanized, and tumors together with main organs (including liver, spleen, kidney, heart, and lung) are harvested. Tumor tissues were accurately weighed and recorded. Subsequently, the tissues were fixed, dehydrated, embedded in paraffin, and sectioned for subsequent histological evaluation. Tissues are labeled by HE for general morphological assessment. Specifically, the paraffin sections were deparaffinized, rehydrated, stained with hematoxylin to label cell nuclei, counterstained with eosin to visualize cytoplasmic structures, and finally mounted for microscopic observation. The detection of apoptotic cells in tumor tissues was carried out using TUNEL. Tissue sections were incubated with the TUNEL reaction mixture for specific labeling of apoptotic cells. Subsequently, the sections were observed under a fluorescence microscope. Additionally, proliferation is assessed via immunohistochemical staining for the proliferation marker Ki67. The number of Ki67‐positive cells was counted to reflect the proliferative capacity of tumor cells. The expression levels of CD24, p21, and CyclinD1 are further examined by immunofluorescence staining. The fluorescence signals were imaged, and the relative expression levels of the target proteins were quantified using Image J software.

### 2.21 Establishment of an Orthotopic Hepatocellular Carcinoma Model and Treatment

To establish the orthotopic liver tumor model in mice, 40 µL of a suspension containing 1 × 10^6^ Hepa1‐6 cells was administered via direct injection beneath the hepatic capsule of the liver lobe. They were assigned to 6 groups (*n* = 5) in a random manner, following the previously mentioned protocol: Control (G1), US (G2), ICG@MSN‐LCD + US (G3), siCD24@MSN‐LCD + US (G4), ICG/siCD24@MSN‐LCD (G5), and ICG/siCD24@MSN‐LCD + US (G6) (ICG dosage: 2 mg/kg, siCD24 dosage: 40 µg/kg). All formulations were administered via tail vein injection at a volume of 100 µL. Mice in the control group received an equal volume of saline through tail vein injection. In the US related‐treated groups, tumors were subjected to US stimulation at 1.5 W/cm^2^ for 2 min post‐injection. All mice were treated on an every‐3‐day schedule (Day 0, 3, 6, and 9). Body weight was also monitored. The experiment was terminated 14 days post‐treatment, and all mice were euthanized. The maximum tumor size was determined by measuring on the liver surface using a vernier caliper, which is commonly used in published orthotopic HCC researches [38, 39, 40, 41]. Consecutive tissue sections were subjected to histological and molecular staining analyses. Routine HE staining was performed for morphological observation. Immunohistochemical staining of Ki67 was applied to evaluate tumor cell proliferation. Finally, immunofluorescence staining was used to detect the expression levels of p21 and Cyclin D1 in tissue samples. The fluorescence signals were imaged, and the relative expression levels of the target proteins were quantified using Image J software.

### 2.22 Statistical Analysis

Experiments were performed at least three times. Statistical analysis was performed according to the Student's *t*‐test or one‐way ANOVA using SPSS software (version 26.0, USA). Significance was expressed as *p < 0.05, **p < 0.01, and ***p < 0.001. The data were presented as mean ± SD.

## 3 Results and Discussion

### 3.1 Preparation and Characterization of ICG/siCD24@MSN‐LCD

**FIGURE 1:**
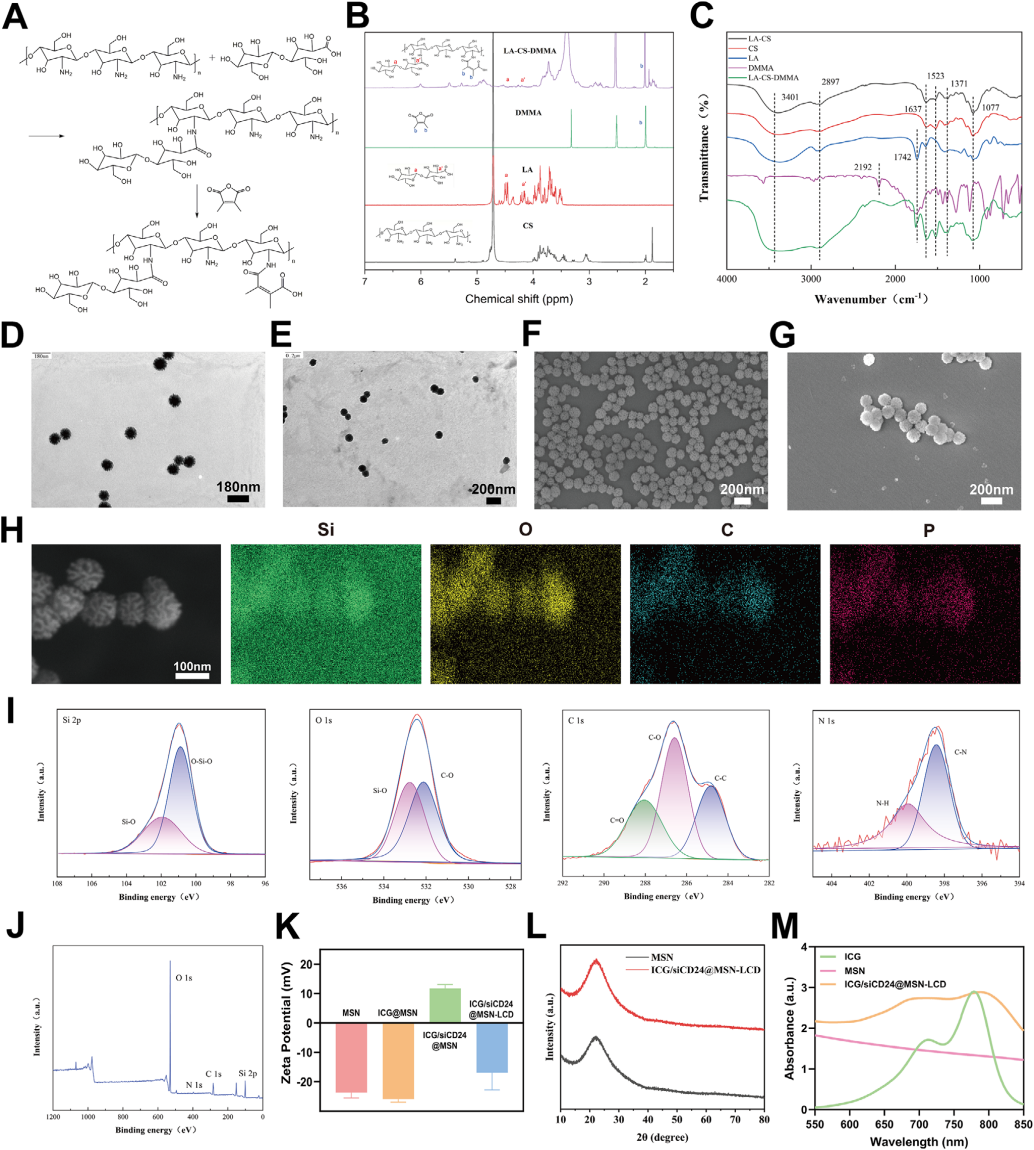
Preparation and characterization of ICG/siCD24@MSN‐LCD. (A) Chemical equation of LCD. (B) ^1^H NMR spectrum of LCD. (C) FT‐IR of LCD. (D) TEM of MSN. Scale bar = 180 nm. (E) TEM of ICG/siCD24@MSN‐LCD. Scale bar = 200 nm. (F) SEM of MSN. Scale bar = 200 nm. (G) SEM of ICG/siCD24@MSN‐LCD. Scale bar = 200 nm. (H) EDS analysis of ICG/siCD24@MSN‐LCD: Si (green), O (yellow), C (blue), and P (purple). Scale bar = 100 nm. (I) XPS spectra of Si 2p, O 1s, C 1s, and N 1s peaks in ICG/siCD24@MSN‐LCD. (J) XPS survey spectrum of ICG/siCD24@MSN‐LCD. (K) Zeta potential of MSN with varying modification degrees. (L) XRD patterns of MSN and ICG/siCD24@MSN‐LCD. (M) UV–vis spectra of MSN, ICG/siCD24@MSN‐LCD, and ICG.

LCD was first synthesized, as illustrated in Figure 1A, and its chemical structure was subsequently confirmed by ^1^H NMR spectrum (Figure 1B) and FT‐IR spectroscopy (Figure 1C). In the ^1^H NMR spectrum of LA, due to the influence of two groups at the ortho position, the signal peaks of acetal hydrogen (a) and hydroxy acid central carbon hydrogen (a') appear near δ 4.2 and 4.5, respectively. The ^1^H NMR spectrum of DMMA shows a singlet peak near δ 2.0, which is assigned to the signal peak of two methyl groups linked to the alkene in the structure (b). In the ^1^H NMR spectrum of LCD, the hydrogen signals of the two methyl groups can be clearly observed, and the characteristic signal peaks of LA can be detected near δ 4.3 and 4.6, indicating the successful preparation of LCD. In addition, as can be seen from the FT‐IR spectra of CS, LA, LA‐CS, DMMA, and their complex LCD, the peak at 2192 cm^−1^ is due to vibration absorption peak of the lactone group in DMMA, and by comparison, it can be seen that this peak disappears in LCD, indicating that the lactone group was consumed by participating in the reaction. The peak at 1742 cm^−1^ corresponds to the stretching vibration of carboxyl group from LA, and comparison shows that this peak disappears in LA‐CS, indicating that the carboxyl group was consumed due to the reaction, but since the reaction between LA‐CS and DMMA regenerates the carboxyl group, this peak reappears in LCD. The peak at 1637 cm^−1^ is the stretching vibration peak of the C═O bond; the peak at 1523 cm^−1^ corresponds to the stretching vibration of the C*─*N bond in CS and LA‐CS; the peak at 1371 cm^−1^ correlates with absorption peak caused by the bending vibration of the N*─*H bond; the peak at 1077 cm^−1^ corresponds to absorption peak caused by stretching vibration of abundant C*─*O bonds, wherein this peak in DMMA is partially shifted due to the influence of C═O bond from lactone group. In summary, LCD was successfully synthesized.

Dynamic light scattering result (Figure S1) indicated a hydrated particle size of 134.7 ± 15.17 nm, and PDI = 0.11 ± 0.07 in MSN. In contrast, the average particle size of ICG/siCD24@MSN‐LCD was measured to be 175.5 ± 3.8 nm, with PDI of 0.18 ± 0.03. TEM and SEM indicated that MSN possess a uniform spherical morphology exhibiting well‐defined mesoporous structure, with clearly observable pore characteristics (Figure 1D,F). In contrast to MSN, the ICG/siCD24@MSN‐LCD exhibited a relatively blurred and less sharp surface mesoporous texture (Figure 1E,G), which may be reasonably attributed to the deposition of a thin LCD layer on the nanoparticle surface. Elemental mapping by EDS confirmed the distribution of silicon (Si) and oxygen (O) in MSN (Figure S2), while additional carbon (C) and phosphorus (P) were clearly observed in ICG/siCD24@MSN‐LCD (Figure 1H). Si and O are primarily derived from the MSN framework, whereas C could originate from diverse sources, including LCD, ICG, and siCD24. Notably, the detected P signal, as a characteristic element of RNA, provides direct evidence for the successful loading of siCD24 onto the MSN. XPS results (Figure S3 and Figure 1I,J) revealed that, compared with MSN, ICG/siCD24@MSN‐LCD exhibited an enhanced C*─*O bond peak intensity, confirming the introduction of organic components. The newly appeared N 1s peak further verified the successful surface modification of LCD. Next, MSN was assessed by N_2_ adsorption–desorption isotherms, which displayed a typical Type IV adsorption isotherm accompanied by a distinct hysteresis loop, characteristic of mesoporous materials (Figure S4A). The average pore diameter was 17.54 ± 0.30 nm, offering sufficient space for the accommodation of ICG. The specific surface area of ICG/siCD24@MSN‐LCD was markedly reduced compared with MSN (Figure S4B), which was attributed to the assembly of siCD24/CS complex on the surface, and subsequent LCD coating on the exterior. To further verify the layer‐by‐layer electrostatic assembly behavior, the surface charge of MSN with different modification degrees was measured. The surface charge of MSN was ‐23.7 ± 1.5 mV. After sequential loading with ICG, adsorption of siCD24/CS complex, and final LCD coating, the corresponding zeta potential changed to ‐25.9 ± 0.8, +11.7 ± 1.1, and ‐16.9 ± 4.8 mV, respectively (Figure 1K). In Figure 1L, XRD patterns of ICG/siCD24@MSN‐LCD are in high agreement with those of MSN. No obvious changes in the characteristic diffraction profiles are observed, indicating that the framework structure of mesoporous silica is well preserved after modification, which is consistent with the literature [42]. As shown in Figure 1M, the UV–vis spectrum of ICG/siCD24@MSN‐LCD exhibits a characteristic absorption peak at 788 nm, which is attributed to ICG. This result further confirmed the successful loading and structural integrity of ICG in the prepared nanoplatform. All the above characterizations collectively confirm the successful fabrication of ICG/siCD24@MSN‐LCD.

### 3.2 Encapsulation, Release, and Ultrasound Imaging of ICG/siCD24@MSN‐LCD

**FIGURE 2:**
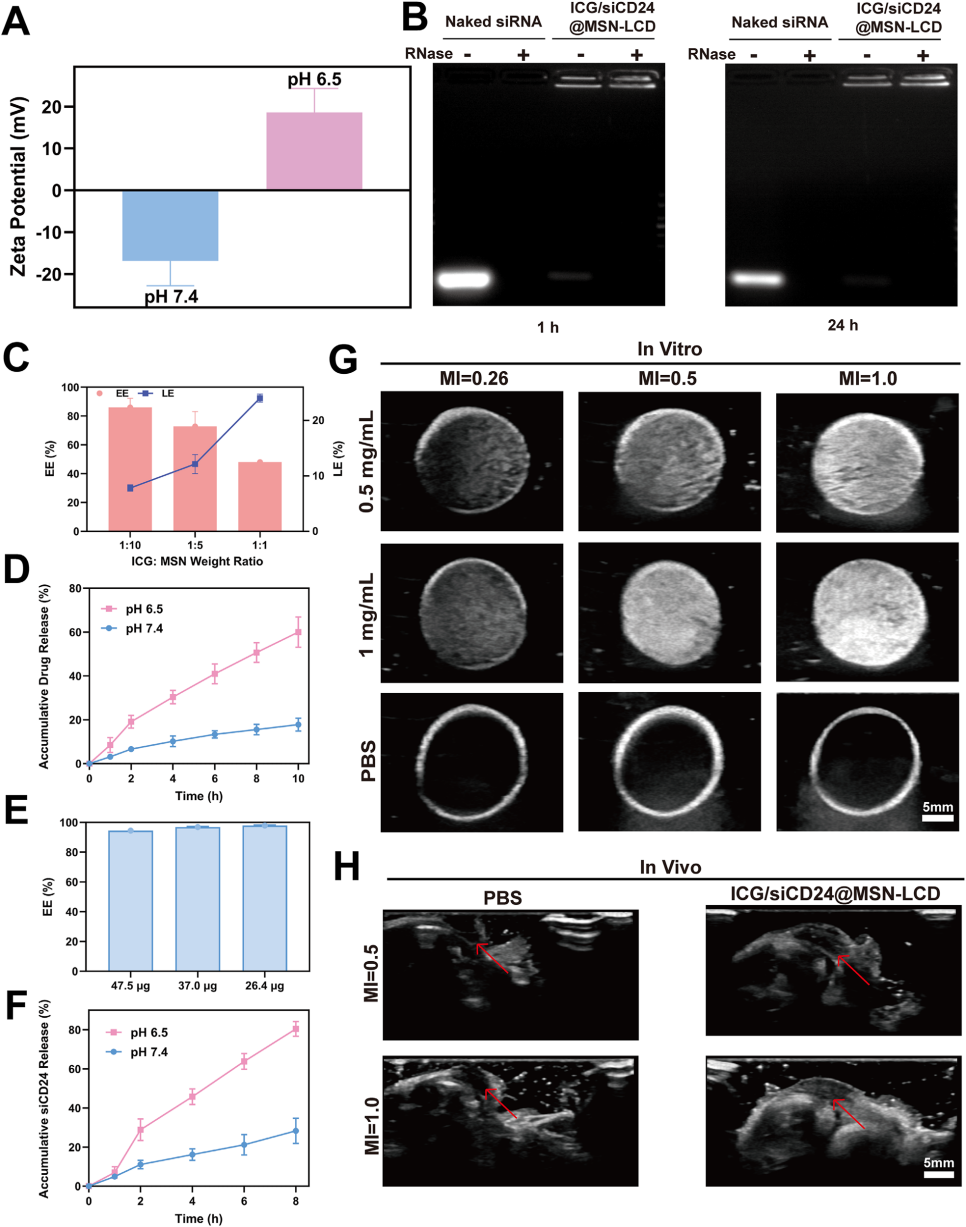
Encapsulation, release, and ultrasound imaging of ICG/siCD24@MSN‐LCD. (A) Zeta potential of ICG/siCD24@MSN‐LCD under different pH conditions. (B) Stability of ICG/siCD24@MSN‐LCD incubated with (+) or without (−) RNase. (C) EE and LE of ICG. (D) Release profile of ICG at different pH values (pH 7.4/6.5). (E) EE of siCD24. (F) Release profile of siCD24 at different pH values (pH 7.4/6.5). (G) In vitro ultrasound imaging. Scale bar = 5 mm. (H) In vivo ultrasound imaging. Scale bar = 5 mm. The red arrows indicate the location of the tumor.

To determine the optimal ICG encapsulation, ICG/siCD24@MSN‐LCD was prepared with varying amounts of ICG. As shown in Figure 2C, the optimal encapsulation was achieved at an ICG: MSN mass ratio of 1:5. At this ratio, the encapsulation efficiency (EE) and loading efficiency (LE) were 72.8% ± 8.4% and 12.1% ± 1.4%, respectively. Meanwhile, the optimal feeding dosage of siCD24 was confirmed to be 26.4 µg, and the encapsulation efficiency was calculated to be 97.86% ± 0.09% (Figure 2E). These two optimal parameters were subsequently used for the preparation of nanoparticles. DMMA bears pH‐responsive functional groups that remain negatively charged under neutral pH conditions, ensuring nanoparticle stability in physiological environments. However, in acidic conditions, DMMA dissociates, leading to a reversal of the nanoparticle charge from negative to positive. Charge conversion capability was assessed by monitoring changes in zeta potential (Figure 2A). At pH 6.5, the zeta potential shifted from ‐16.9 ± 4.8 to +18.6 ± 4.7 mV, confirming the charge reversal behavior of ICG/siCD24@MSN‐LCD in an acidic condition. Due to the pH‐responsive nature of ICG/siCD24@MSN‐LCD, its surface charge becomes positive at an acidic microenvironment, causing repulsion between the positively charged outer LCD coating and the internally positively charged ICG/siCD24@MSN, which promotes the detachment of the LCD layer. After the removal of the LCD coating, the siCD24/CS complex is directly exposed on the surface. Once internalized into the cytoplasm, siCD24 gradually dissociates from CS, and this process can be reasonably explained by three factors: the intracellular electrostatic shielding effect [43], the decreased protonation degree of CS under neutral pH [44], and the competitive binding with negatively charged endogenous biomacromolecules [45]. Along with the gradual disassembly of the surface siCD24/CS complex, sustained diffusion and release of both siCD24 and encapsulated ICG can be achieved. Therefore, we investigated the in vitro release profiles of ICG (Figure 2D) and siCD24 (Figure 2F) from ICG/siCD24@MSN‐LCD under different pH conditions. Within 10 h, the cumulative release of ICG was only 17.8% ± 2.4% at pH 7.4, while it increased prominently to 60.0% ± 5.6% at pH 6.5. Similarly, after 8 h of incubation, the siCD24 release rate reached 80.4% ± 3.7% at pH 6.5, whereas only 28.3% ± 6.4% was released at pH 7.4. Collectively, the release results revealed that ICG/siCD24@MSN‐LCD possessed prominent pH‐sensitive release property, enabling rapid payload release in the tumor.

To clarify the CD24 expression in HCC, immunohistochemical staining on human hepatocellular carcinoma tissues was conducted. The results revealed a significantly higher level of CD24 expression in HCC tumor tissues compared to nontumoral tissues (Figure S5). On this basis, ICG/siCD24@MSN‐LCD was constructed to achieve CD24 silencing. We first investigated the gene knockdown efficiency of the loaded siCD24, as demonstrated in Figure S6, the ICG/siCD24@MSN‐LCD group exhibited significantly higher silencing efficiency compared to control group and siNC group (*p < 0.05), with no statistically significant difference observed relative to free siCD24 transfection group. Considering that naked siRNA is vulnerable to degradation by RNase in the physiological microenvironment, we further evaluated the protective stability of siCD24 encapsulated in ICG/siCD24@MSN‐LCD. To verify this point, we incubated the different samples with RNase prior to electrophoresis. As shown in Figure 2B, the nanoparticle‐encapsulated siRNA did not migrate through the agarose gel, whereas the naked siRNA migrated freely. Notably, the naked siRNA was degraded after just 1 h of RNase incubation, while the encapsulated siRNA remained intact even after 24 h. These results indicate that ICG/siCD24@MSN‐LCD provides efficient loading and ideal protection for siRNA, demonstrating their excellent stability as a delivery vehicle.

Biocompatibility was assessed through hemolysis assays using red blood cells and Calcein‐AM/PI double staining using Huh7 cells, both incubated with MSN (Figures S7 and S8). The MSN exhibited minimal hemolysis (3.56% ± 0.75%) even at the highest concentration tested, which is well below the accepted safety threshold [46], further supported by absence of cell death in Huh7 cells in Calcein‐AM/PI assay. The US imaging capability of ICG/siCD24@MSN‐LCD was also explored. The nanoparticles showed enhanced B‐mode signals, and the signal intensity increased with nanoparticle concentration and MI (Figure 2G). In tumor‐bearing mice, the ICG/siCD24@MSN‐LCD group exhibited significantly stronger B‐mode signals at the tumor site compared with the PBS control group (Figure 2H), indicating that ICG/siCD24@MSN‐LCD effectively enhances ultrasound backscattering, thereby improving contrast in conventional US imaging. More importantly, with increasing MI, the ultrasound imaging performance improved correspondingly. The results indicate that ICG/siCD24@MSN‐LCD exhibits strong ultrasound imaging capability, highlighting its potential as a theranostic agent for determining tumor size and location, as well as monitoring therapeutic efficacy.

### 3.3 Cellular Adhesion and Biodistribution

**FIGURE 3:**
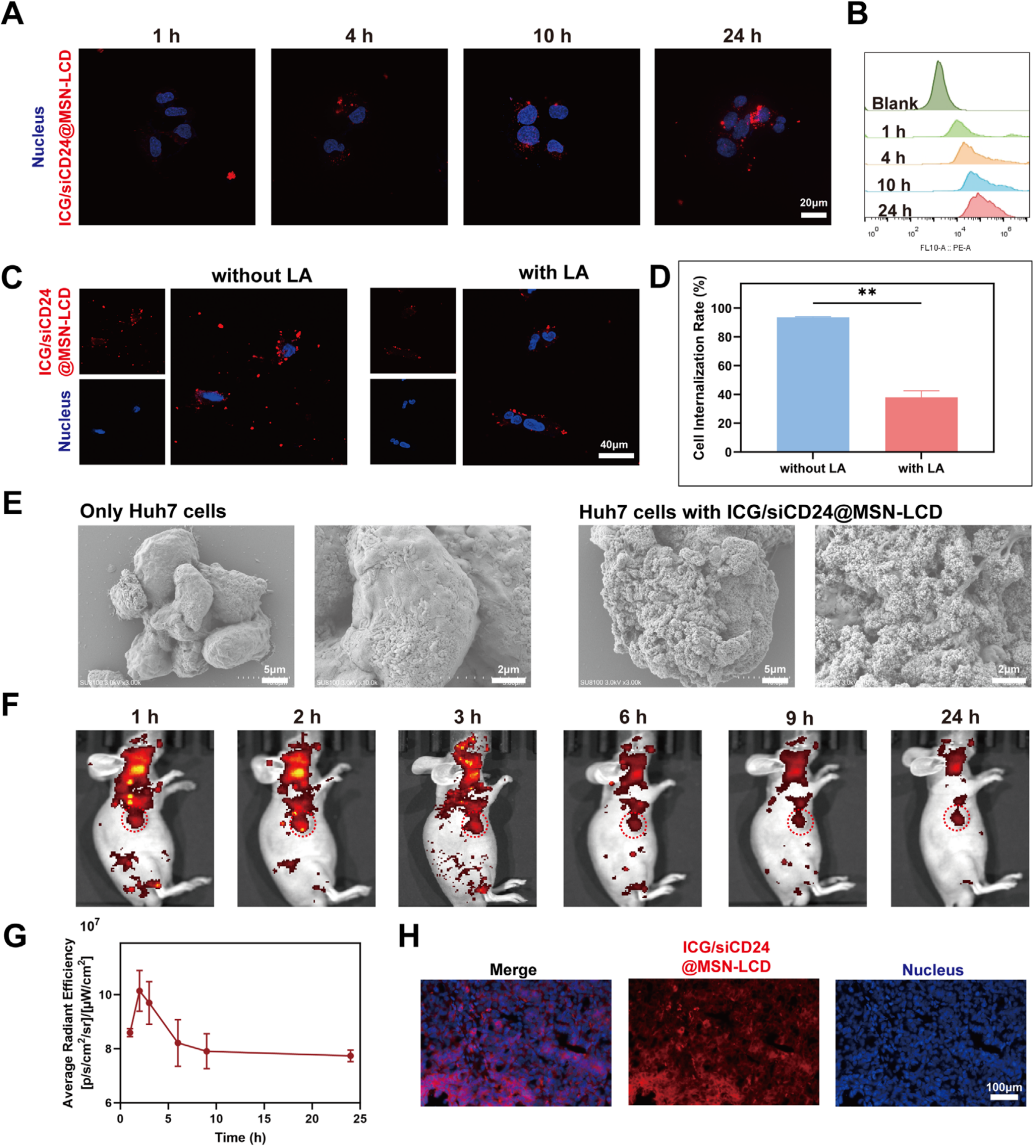
Cellular adhesion and biodistribution. (A) CLSM images of cellular adhesion of ICG/siCD24@MSN‐LCD by Huh7 cells. Scale bar = 20 µm. (B) FCM analysis of cellular adhesion of ICG/siCD24@MSN‐LCD by Huh7 cells. (C) CLSM images of cellular adhesion of ICG/siCD24@MSN‐LCD by Huh7 cells with/without LA. Scale bar = 40 µm. (D) Cell internalization rate of ICG/siCD24@MSN‐LCD obtained from FCM. (E) SEM images of Huh7 cells with/without ICG/siCD24@MSN‐LCD. Scale bars = 5 µm, 2 µm. (F) Representative images and (G) quantification at selected time points post‐injection. The red circles indicate the location of the tumor. (H) Fluorescence images of penetration of ICG/siCD24@MSN‐LCD at tumor. Scale bar = 100 µm.

Effective tumor targeting is essential for achieving therapeutic efficacy. In this study, LA was conjugated to achieve active targeting. To investigate the targeting capability and cellular uptake of the ICG/siCD24@MSN‐LCD, Huh7 cells were incubated with ICG/siCD24@MSN‐LCD for different durations and then subjected to CLSM imaging (Figure 3A). As the incubation time increased, cellular uptake of the nanoparticles (red dots) also increased, indicating that the internalization process is time‐dependent. Furthermore, FCM was employed to quantitatively evaluate the endocytic capacity of Huh7 cells after incubation with ICG/siCD24@MSN‐LCD for various time periods (Figure 3B and Figure S9). The results were in good agreement with the CLSM. To directly visualize the interaction between cells and the ICG/siCD24@MSN‐LCD, the surface morphology of Huh7 cells incubated with or without ICG/siCD24@MSN‐LCD was examined using SEM. Compared to the untreated Huh7 cells, surfaces of cells incubated with nanoparticles showed substantial adhesion and aggregation of nanoparticles (Figure 3E), further confirming strong cellular adhesion.

The ASGPR, which is highly expressed in HCC cells, garnered significant interest for targeting liver tumors. It specifically interacts with the galactose moiety or *N*‐acetylgalactosamine terminal residues of desialylated glycoproteins. The affinity increases with the valence of the sugar residues—a process termed the cluster glycoside effect [47, 48, 49]. To explore whether cell adhesion of ICG/siCD24@MSN‐LCD is mediated by specific recognition between LA and ASGPR, a competition assay was conducted with free LA. Results showed that cell uptake of ICG/siCD24@MSN‐LCD + LA was lower than that of ICG/siCD24@MSN‐LCD (Figure 3C). This reduction is likely attributable to free LA competing with the ICG/siCD24@MSN‐LCD for binding to ASGPR. These findings were further confirmed by FCM (Figure S10 and Figure 3D).

To examine in vivo biodistribution of ICG/siCD24@MSN‐LCD, the Rhodamine B‐labeled ICG/siCD24@MSN‐LCD was intravenously administered to nude mice. As shown in Figure 3F,G, a time‐dependent increase in fluorescence signal intensity was noted, reaching its peak at 2 h post‐injection, and subsequently decreased gradually in the following hours. In addition, we further investigated the in vivo biodistribution. The results are shown in Figure S11. At 2 h after administration, the fluorescence signal was the strongest in tumor tissue, which was attributed to active targeting accumulation mediated by lactobionic acid. Obvious signals were also observed in the liver and kidney, resulting from capture by the hepatic reticuloendothelial system and renal excretion. At 24 h, the overall fluorescence intensity decreased gradually with metabolism, while fluorescence signals were still detectable in tumor tissue, suggesting effective retention of nanoparticles at the tumor site. Furthermore, the efficient accumulation of ICG/siCD24@MSN‐LCD in tumor tissues was visualized in frozen sections (Figure 3H). This observation indicates that ICG/siCD24@MSN‐LCD possesses the ability for prolonged accumulation and maintained stability within the tumor tissue. Furthermore, key serum biochemical parameters in mice were evaluated to assess potential adverse effects. As shown in Figure S12, ALT, AST, BUN, and CREA remained within reference intervals comparable to control group, indicating that our nanoparticles exhibited excellent biocompatibility, rendering them highly suitable for application as drug delivery systems.

### 3.4 SDT‐Induced Free Radical Generation and RNA Sequencing Analysis

**FIGURE 4:**
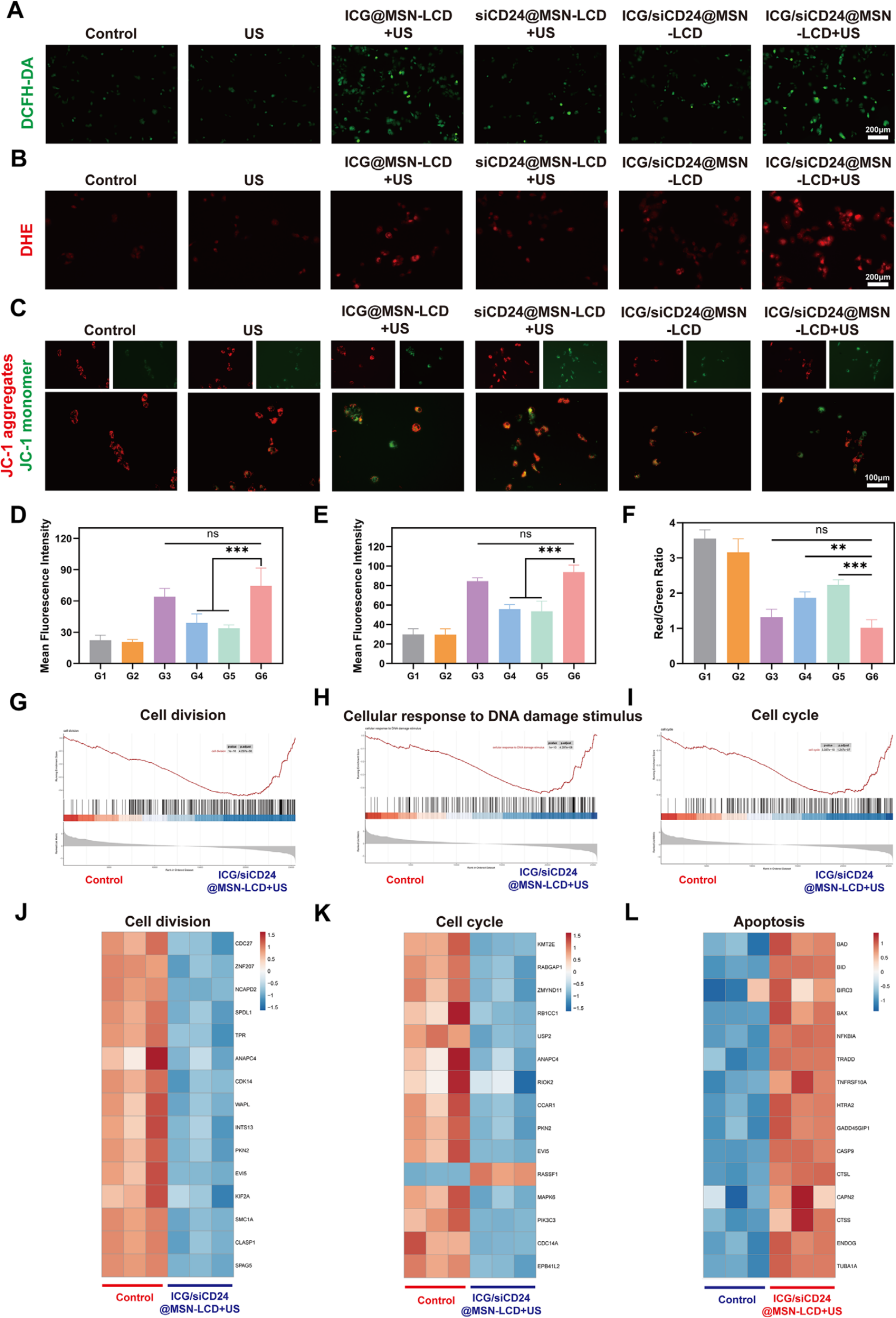
SDT‐induced free radical generation and RNA sequencing analysis. (A) DCFH‐DA staining in Huh7 cells. Scale bar = 200 µm. (B) DHE staining in Huh7 cells. Scale bar = 200 µm. (C) JC‐1 staining in Huh7 cells. Scale bar = 100 µm. (D) Quantitative analysis of ROS in Huh7 cells from A. (E) Quantitative analysis of DHE in Huh7 cells from B. (F) Quantitative analysis of JC‐1 in Huh7 cells from C. (G) GSEA reveals enrichment of the “cell division” pathway in Huh7 cells. (H) GSEA reveals enrichment of the “cellular response to DNA damage stimulus” pathway in Huh7 cells. (I) GSEA reveals enrichment of the “cell cycle” pathway in Huh7 cells. (J) Heatmap of genes associated with cell division from Huh7 cell transcriptome sequencing. (K) Heatmap of cell cycle‐related genes from Huh7 cell transcriptome sequencing. (L) Heatmap of apoptosis‐associated genes from Huh7 cell transcriptome sequencing.

Numerous studies have demonstrated that under ultrasound irradiation, ICG can function as a sonosensitizer by generating ROS, resulting in potent cytotoxic effects [50, 51]. Therefore, sonosensitizing capability of ICG and its potential in SDT were evaluated. Cellular ROS was first explored through DCFH‐DA. Compared with control group, cells treated with ICG@MSN‐LCD or ICG/siCD24@MSN‐LCD groups containing ICG exhibited a marked increase in green fluorescence under fluorescence microscopy following ultrasound irradiation, with no statistically significant difference observed between the two ICG‐treated groups (Figure 4A,D). The results confirm that ICG‐loaded nanoparticles can effectively trigger an intracellular burst of endogenous ROS under ultrasound exposure. The ICG/siCD24@MSN‐LCD+US group exhibited a significant difference relative to the siCD24@MSN‐LCD+US group lacking ICG (***p < 0.001); cells co‐cultured with ICG/siCD24@MSN‐LCD produced minimal endogenous ROS in the absence of ultrasound irradiation. In contrast, a marked increase in ROS levels was observed upon ultrasound exposure (***p < 0.001), which further verified that the robust ROS generation in the ICG/siCD24@MSN‐LCD+US group originated from the combined effect of ICG and ultrasound irradiation, rather than other intrinsic factors of the nanoparticles alone, consistent with previous reports [52].

DHE is widely used to detect intracellular superoxide radicals [53]. In its reduced state, DHE exhibits blue fluorescence. Upon oxidation by superoxide, it is converted to 2‐hydroxyethidium, a product that intercalates with DNA and displays intense red fluorescence. Due to its high membrane permeability and compatibility with standard fluorescence microscopy, DHE offers a convenient and accessible method for monitoring dynamic changes in reactive oxygen species within living cells. Consistent with the DCFH‐DA result, cells treated with ICG@MSN‐LCD+US or ICG/siCD24@MSN‐LCD+US exhibited intense red fluorescence (Figure 4B,E), with no significant difference between the two ICG‐containing groups. In contrast, both the siCD24@MSN‐LCD+US group and the ICG/siCD24@MSN‐LCD group showed markedly lower DHE levels than the ICG/siCD24@MSN‐LCD+US group (***p < 0.001). This discovery reveals that combined treatment initiates a more complex ROS cascade effect.

The overproduction of ROS is one of the primary mechanisms underlying mitochondrial damage [54]. Therefore, we assessed mitochondrial function using JC‐1 staining. Under normal physiological conditions, JC‐1 readily enters mitochondria and forms aggregates (red fluorescence); under damaging conditions, however, it remains in monomeric form (green fluorescence), indicating mitochondrial dysfunction. As shown in Figure 4C,F, both the ICG@MSN‐LCD+US and ICG/siCD24@MSN‐LCD+US groups exhibited a sharp decrease in the JC‐1 red/green fluorescence ratio, suggesting significant mitochondrial dysfunction. No significant difference was observed between the two ICG‐treated groups. Consistent with the ROS and DHE results above, mitochondrial damage was specifically initiated by the combination of ICG‐mediated sonosensitization and ultrasonic irradiation, which is in line with the previously established mechanism of SDT therapy‐induced mitochondrial injury [55].

To elucidate the mechanism underlying the potent antitumor efficacy of ICG/siCD24@MSN‐LCD combined with US, transcriptomic profiling of treated Huh7 cells was conducted by RNA sequencing. Comparative analysis revealed 4334 significantly down‐regulated (green) and 3919 up‐regulated (red) genes compared with control group (Figure S13A). GO enrichment analysis identified that ICG/siCD24@MSN‐LCD‐mediated sonogenetic therapy primarily affected biological processes and molecular functions including cell division, cellular response to DNA damage stimulus, cell cycle, ATP binding, and DNA binding (Figure S13B). GSEA further confirmed that the observed trends in cell division, cellular response to DNA damage stimulus, and cell cycle progression are consistent with GO results (Figure 4G–I). Collectively, these results demonstrate that ICG/siCD24@MSN‐LCD‐based sonogenetic therapy disrupts essential cellular functions and impedes vital processes in Huh7 cells, contributing to its therapeutic outcome. Heatmap analysis (Figure 4J) revealed the significant alterations in the cell division pathway in the ICG/siCD24@MSN‐LCD‐mediated sonogenetic therapy group. Specifically, CDC27 disrupts mitotic progression and genomic stability by regulating APC/C complex‐mediated cyclin degradation [56]; SMC1A, as a core component of the cohesin complex, causes chromosome segregation errors when dysregulated [57]; and SPAG5 promotes tumor proliferation by interfering with the spindle assembly checkpoint [58]. Besides, ICG/siCD24@MSN‐LCD‐mediated sonogenetic therapy not only induced significant disruption in cell division but also specifically suppressed cell cycle progression (Figure 4K). Mechanistically, downregulation of USP2 accelerated Cyclin D1 degradation, triggering G1/S phase arrest, whereas RB1CC1 expression induced RB1, particularly its underphosphorylated forms, resulting in cell cycle arrest in neoplastic cells [59, 60, 61]. Furthermore, transcriptomic analysis confirmed that the combination therapy successfully activated the programmed cell death pathway (Figure 4L). Specifically, the core executioners BAX and BID were cooperatively activated, directly mediating the irreversible loss of mitochondrial membrane permeability [62, 63]. Concurrently, upregulation of BAD relieved intrinsic inhibition of apoptosis, thereby indirectly promoting the initiation of the mitochondrial apoptosis pathway [64]. These results demonstrate that the ICG/siCD24@MSN‐LCD‐mediated sonogenetic therapy modulates multiple biological processes and signaling pathways, including but not limited to cell division, apoptosis, and cell cycle regulation, ultimately leading to cancer cell death.

### 3.5 Activation of the p53‐Related Pathway by Synergistic Sono‐Gene Therapy

**FIGURE 5:**
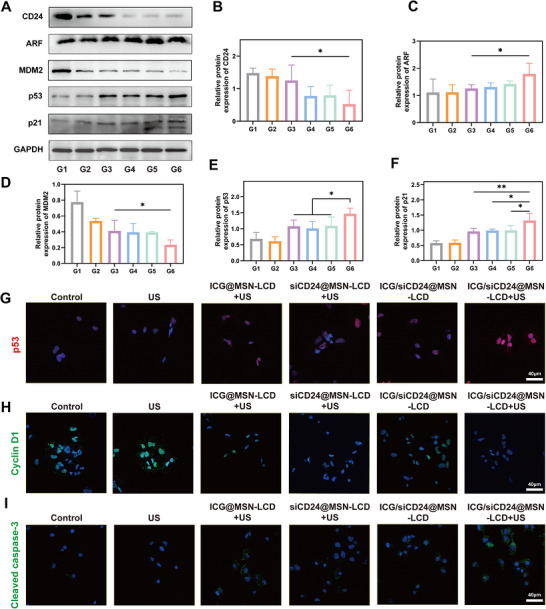
Activation of the p53‐related pathway by synergistic sono‐gene therapy. (A)Western blot images of pathway proteins. (B) Relative expression level of CD24. (C) Relative expression level of ARF. (D) Relative expression level of MDM2. (E) Relative expression level of p53. (F) Relative expression level of p21. (G) Immunofluorescence images of p53 in Huh7 cells. Scale bar = 40 µm. (H) Immunofluorescence images of Cyclin D1 in Huh7 cells. Scale bar = 40 µm. (I) Representative immunofluorescence images of Cleaved caspase‐3 expression after different treatments in Huh7 cells. Scale bar = 40 µm.

Previous studies have demonstrated that CD24 can competitively inhibit the binding of ARF to NPM, resulting in structural instability of ARF, which in turn upregulates MDM2 expression and ultimately reduces the levels of p53 and its key downstream target p21/CDKN1A. This further enhances the kinase activity of the Cyclin D1‐CDK4/6 complex, significantly promoting tumor initiation, progression, and therapy resistance [65]. Therefore, we investigated whether ICG/siCD24@MSN‐LCD acts on this pathway by silencing CD24 to suppress tumor progression. The expressions of CD24 and MDM2 were significantly downregulated in the ICG/siCD24@MSN‐LCD+US group, while the expressions of ARF, p53, and p21 were markedly upregulated (Figure 5A). The relative expression levels of each protein are displayed in Figure 5B–F, with results consistent with the aforementioned findings. The expression of CD24, ARF, and MDM2 in the ICG/siCD24@MSN‐LCD+US group showed no significant differences compared to the siCD24@MSN‐LCD+US and ICG/siCD24@MSN‐LCD groups, leading to the reasonable inference that the siCD24 loaded within the nanoparticles induced a series of molecular changes. Notably, the expression levels of p53 and p21 in the ICG/siCD24@MSN‐LCD+US group were significantly higher than those in the other treatment groups, demonstrating that ICG also played an important role in activating the p53‐related pathway, and its combination with siCD24 could produce a synergistic amplification effect.

To further verify the expression change of CD24 and the knockdown effect of siCD24 at the cellular level, cellular immunofluorescence staining of CD24 was performed (Figure S14). Consistent with the Western blot results, the fluorescence intensity of CD24 was significantly decreased in the ICG/siCD24@MSN‐LCD+US group relative to the ICG@MSN‐LCD+US group (**p < 0.01), whereas no obvious statistical difference in CD24 expression was observed among siCD24@MSN‐LCD+US, ICG/siCD24@MSN‐LCD, and ICG/siCD24@MSN‐LCD+US groups, which further verified the knockdown effect of siCD24 delivered by nanoparticles. Subsequently, cell immunofluorescence was conducted to detect the expression of p53. The red fluorescence representing p53 protein was weak in both the control and ultrasound‐only groups (Figure 5G). In contrast, treatment with ICG/siCD24@MSN‐LCD+US resulted in a significant enhancement of p53 fluorescence intensity in cell nuclei relative to other groups (***p < 0.001) (Figure S15), further suggesting that the robust activation of p53 was attributed to the synergistic effect of ICG‐mediated SDT and siCD24 gene silencing under ultrasound irradiation. To further explore the underlying mechanism by which ICG regulates p53, we treated cells with NAC to inhibit intracellular ROS production and subsequently detected the p53 expression level (Figure S16). The results revealed that ICG@MSN‐LCD+US significantly upregulated p53 expression, whereas NAC intervention markedly reversed this trend (***p < 0.001). Mechanistically, ICG‐mediated SDT elevates intracellular ROS levels to trigger oxidative stress, which further upregulates p53 and initiates tumor cell apoptosis, consistent with previous reports [66, 67]. Collectively, these findings demonstrate that ICG/siCD24@MSN‐LCD‐mediated sono‐gene therapy activates the p53 signaling pathway through dual mechanisms: CD24 silencing via siCD24 and oxidative stress activation via ICG‐induced ROS production.

Next, we assessed the expression of p53‐related effector proteins by Western blot and immunofluorescence. Cyclin D1 is a key regulator of the cell cycle and plays a critical role in the G1‐S phase transition [68]. Its accumulation can lead to uncontrolled cell cycle progression and accelerated cell division, ultimately resulting in tumor progression. p53 can inhibit the activity of CDK4/6 by activating p21, thereby preventing the Cyclin D1‐CDK4/6 complex from phosphorylating the Rb protein [69]. Then, we examined the expression of p53 downstream molecules: p21 and Cyclin D1.

As shown in Figure S17, the green fluorescence intensity of p21 was markedly upregulated in the ICG/siCD24@MSN‐LCD+US group (**p < 0.01 vs. ICG@MSN‐LCD+US and siCD24@MSN‐LCD+US; *p < 0.05 vs. ICG/siCD24@MSN‐LCD). In contrast, Cyclin D1 showed the opposite expression trend, with significant differences observed in the ICG/siCD24@MSN‐LCD+US group compared with the ICG@MSN‐LCD+US, siCD24@MSN‐LCD+US, and ICG/siCD24@MSN‐LCD groups (**p < 0.01) (Figure 5H and Figure S18). These results were consistent with those of Western blot analysis, further demonstrating that ICG/siCD24@MSN‐LCD combined with ultrasound treatment could block the cell cycle of Huh7 cells.

Caspase‐3 is the most crucial apoptosis‐regulating protein and becomes activated (generating Cleaved caspase‐3) during apoptosis [70]. In Figure 5I and Figure S19, the expression of Cleaved caspase‐3 was significantly higher in the ICG/siCD24@MSN‐LCD+US group (***p < 0.001 vs. ICG@MSN‐LCD+US; **p < 0.01 vs. siCD24@MSN‐LCD+US and ICG/siCD24@MSN‐LCD), proving that the combined therapy could more substantially upregulate the expression of apoptosis‐related proteins.

### 3.6 In Vitro Therapeutic Efficacy

**FIGURE 6:**
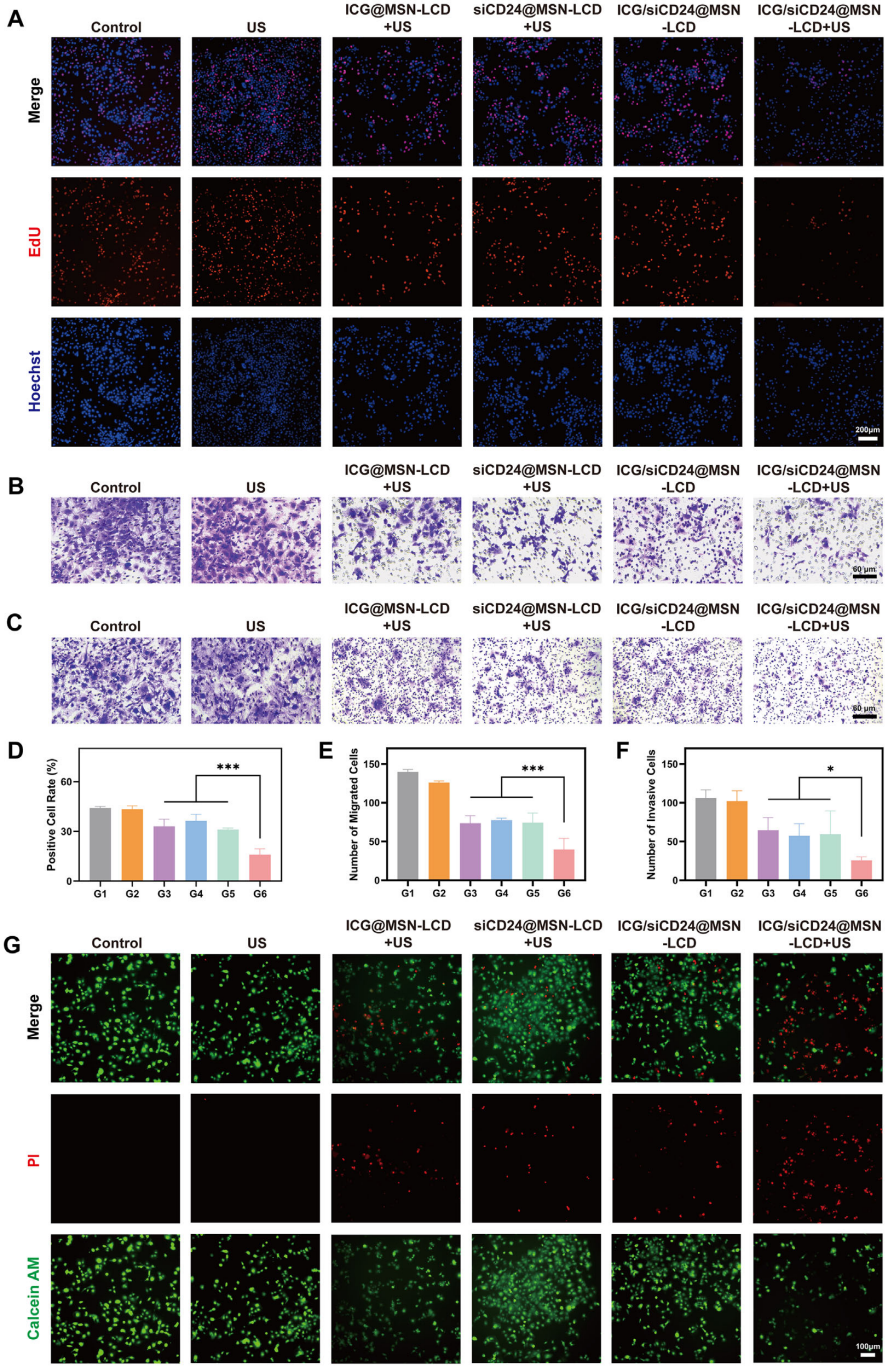
In vitro therapeutic efficacy. (A) Fluorescence image of EdU staining in Huh7 cells. Scale bar = 200 µm. (B) Crystal violet staining of Huh7 cell migration. Scale bar = 60 µm. (C) Crystal violet staining of Huh7 cell invasion. Scale bar = 60 µm. (D) Quantification of EdU‐positive cells. (E) Number of migrated cells. (F) Number of invaded cells. (G) Fluorescence image of live/dead staining in Huh7 cells. Scale bar = 100 µm.

We next evaluated the therapeutic efficacy of ICG/siCD24@MSN‐LCD combined with ultrasound stimulation against Huh7 cells in vitro. Cell proliferation was assessed using EdU assay, with results presented in Figure 6A,D. Proliferating cells were labeled red (EdU‐positive) against blue‐stained nuclei. Abundant proliferating cells were detected under only ultrasound, suggesting its limited suppressive effect. The ICG/siCD24@MSN‐LCD+US group showed significantly stronger inhibition of cell proliferation (16.0% ± 2.8% vs. 31.0% ± 0.8%) compared to ICG/siCD24@MSN‐LCD without ultrasound, highlighting the essential role of ultrasound irradiation in the therapeutic mechanism. Furthermore, the ICG/siCD24@MSN‐LCD+US group exhibited superior anti‐proliferative effects compared to both ICG@MSN‐LCD+US (33.0% ± 3.6%) and siCD24@MSN‐LCD+US (36.3% ± 3.3%) groups, confirming the enhanced efficacy of the combined sonogenetic approach over individual monotherapies.

Subsequently, we further assessed tumor cell apoptosis. TUNEL staining revealed that ICG/siCD24@MSN‐LCD+US treatment (***p < 0.001 vs. ICG@MSN‐LCD+US, siCD24@MSN‐LCD+US; **p < 0.01 vs. ICG/siCD24@MSN‐LCD) significantly enhanced tumor cell apoptosis (Figure S20), indicating that the combined therapy exerted its antitumor activity via inducing cell apoptosis.

Migration and invasion capabilities were determined by Transwell assays. The number of migrating cells in the ICG/siCD24@MSN‐LCD+US group (40±12) was significantly lower than those in ICG@MSN‐LCD+US (74±8), siCD24@MSN‐LCD+US (78±2), and ICG/siCD24@MSN‐LCD (74±10) groups (Figure 6B,E). Assessment of invasive potential (Figure 6C,d,F) revealed substantially more invading cells in control and ultrasound‐only groups compared to all treatment groups. Among the therapeutic groups, the ICG/siCD24@MSN‐LCD+US group showed the fewest invading cells (26±4), significantly less than those in ICG@MSN‐LCD+US (65±13), siCD24@MSN‐LCD+US (57±13), and ICG/siCD24@MSN‐LCD (59±24) groups. These results collectively demonstrate the superior effectiveness of ICG/siCD24@MSN‐LCD under ultrasound irradiation in suppressing both migration and invasion of Huh7 cells.

Calcein‐AM/PI double staining was used to evaluate cell viability and toxicity. In Figure 6G, while virtually no red‐stained necrotic cells were observed in the control and ultrasound‐only groups, all treatments indicated different degrees of necrosis. ICG/siCD24@MSN‐LCD+US group induced the most pronounced cytotoxic effect, as evidenced by the highest level of red‐fluorescent necrotic cells. Based on the collective evidence, the combination of ICG/siCD24@MSN‐LCD with ultrasound irradiation produces a synergistic anti‐tumor response, characterized by concurrent suppression of proliferation, induction of apoptosis, impairment of migratory and invasive capabilities, and induction of cell death.

### 3.7 Therapeutic Efficacy in Subcutaneous Models

**FIGURE 7:**
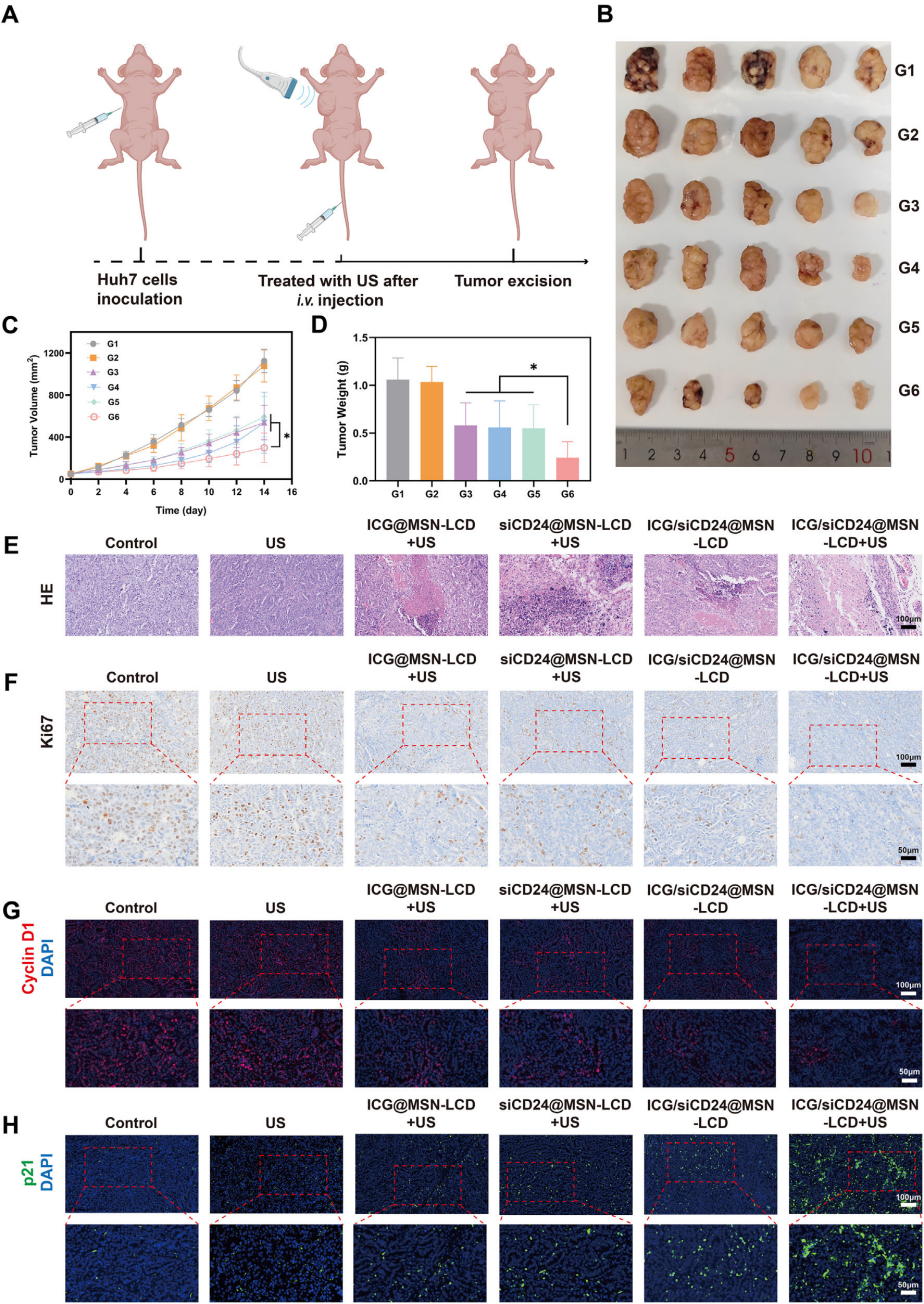
Therapeutic efficacy in subcutaneous models. (A) Schematic diagram of establishing and treating subcutaneous tumor model in nude mice using Huh7 cells. *n* = 5. (B) Photograph of subcutaneous tumors. (C) Tumor volume changes at various treatment groups. (D) Tumor weight at various treatment groups. (E) HE staining of subcutaneous tumors across different treatment groups. Scale bar = 100 µm. (F) Immunohistochemical staining of Ki67 in tumors across various treatment groups. Scale bars = 100, 50 µm. (G) Immunofluorescence staining of Cyclin D1 in tumors across various treatment groups. Scale bars = 100, 50 µm. (H) Immunofluorescence staining of p21 in tumors across various treatment groups. Scale bars = 100, 50 µm.

Encouraged by the promising in vitro results, we proceeded to evaluate the therapeutic efficacy of ICG/siCD24@MSN‐LCD combined with ultrasound in vivo. A human hepatocellular carcinoma xenograft model was established in nude mice using Huh7 cells. All treatments were administered according to the experimental schedule (Figure 7A), with tumor volume and body weight recorded every two days. At day 14, nude mice were euthanized, and tumors were excised. Representative tumor images are shown in Figure 7B, and tumor weights measured on day 14 are summarized in Figure 7D. Moderate tumor growth inhibition was observed in the ICG@MSN‐LCD+US, siCD24@MSN‐LCD+US, and ICG/siCD24@MSN‐LCD groups. The ICG/siCD24@MSN‐LCD+US group displayed the most obvious tumor suppression, with a significantly slower growth rate and the smallest final tumor volume among all groups (Figure 7C), indicating that the sonogenetic combination therapy plays a predominant role in tumor suppression. Tumor weight further supported these findings. After 14 days of treatment, tumor weights in the ICG@MSN‐LCD+US (0.58 ± 0.21 g), siCD24@MSN‐LCD+US (0.56 ± 0.25 g), ICG/siCD24@MSN‐LCD (0.55 ± 0.22 g), and ICG/siCD24@MSN‐LCD+US (0.24 ± 0.15 g) groups were obviously lower than those in Control and US groups. ICG/siCD24@MSN‐LCD+US group demonstrated the most potent antitumor efficacy, as evidenced by the consistently smallest tumor weight. Additionally, no significant differences in body weight were observed among any experimental groups compared to the Control group (Figure S21), suggesting the absence of overt systemic toxicity during the treatment period.

To elucidate the potential mechanisms underlying tumor growth inhibition, histological analysis of tumor tissues was performed using HE, TUNEL, Ki67 immunohistochemistry, and immunofluorescence staining for cell cycle indicators. The results revealed that the ICG/siCD24@MSN‐LCD+US group exhibited extensive necrotic areas with significant nuclear loss (Figure 7E), an obvious increase in TUNEL‐positive apoptotic cells (Figure S22), a marked reduction in Ki67‐positive proliferating cells (Figure 7F and Figure S23), substantially decreased CD24 (Figure S24) and Cyclin D1 expression (Figure 7G and Figure S25), and notably enhanced p21 expression (Figure 7H and Figure S26). These results collectively demonstrate that ICG/siCD24@MSN‐LCD combined with ultrasound amplifies antitumor efficacy through simultaneous ultrasound activation of the sonosensitizer ICG and CD24 silencing by siCD24. Furthermore, to evaluate the biosafety of the different treatments, histological examination of major organs from nude mice in each treatment group was conducted via HE staining. In Figure S27, no significant pathological abnormalities were found within these organs, indicating the high biocompatibility of ICG/siCD24@MSN‐LCD+US treatment.

### 3.8 Therapeutic Efficacy in Orthotopic Models

**FIGURE 8:**
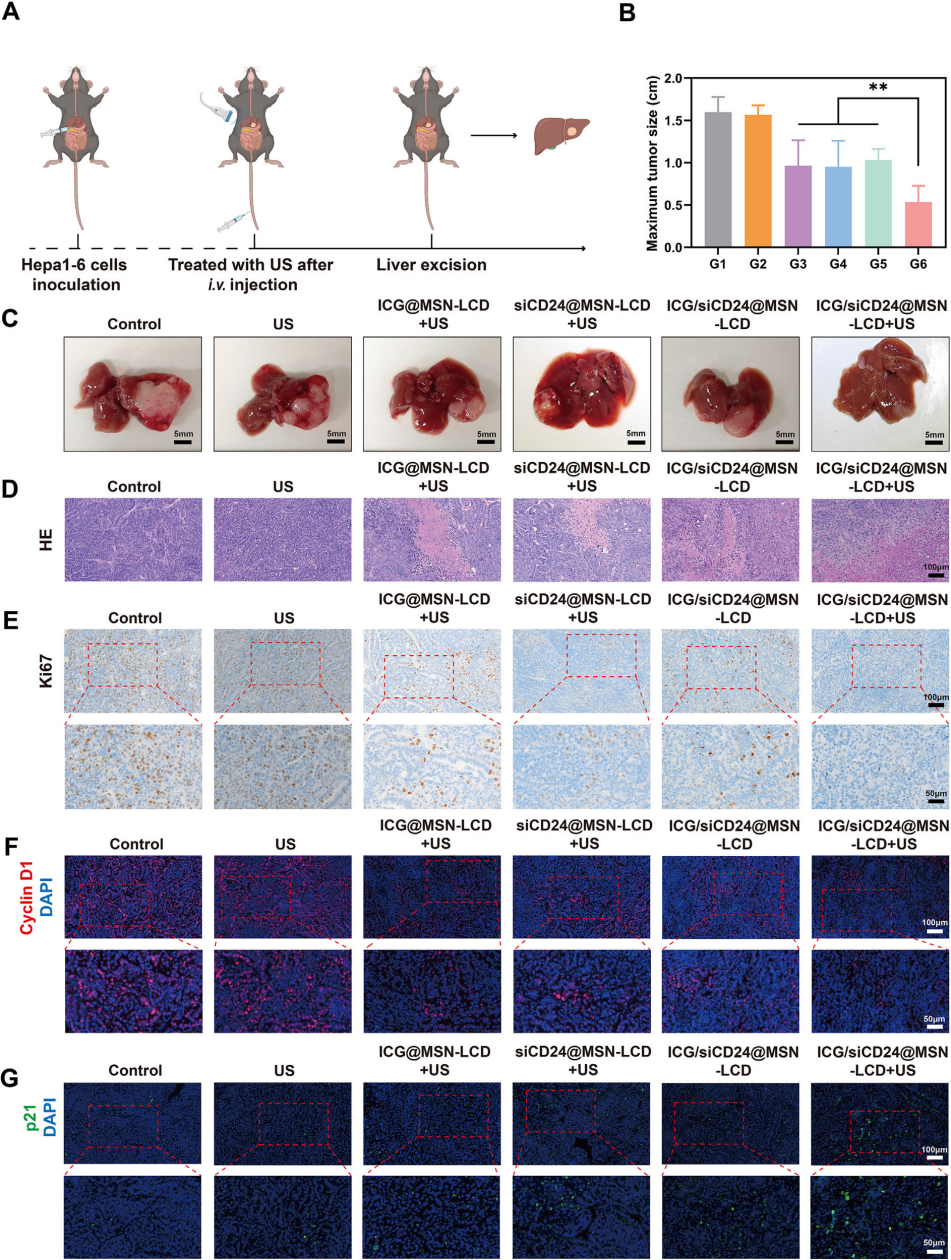
Therapeutic efficacy in orthotopic models. (A) Schematic diagram of establishing and treating orthotopic tumor models in C57 mice using Hepa1‐6 cells. (B) Maximum tumor size in different treatment groups of orthotopic models. *n* = 5. (C) Representative images of orthotopic tumors across treatment groups. (D) HE staining of orthotopic tumor tissues. Scale bar = 100 µm. (E) Immunohistochemical staining of Ki67. Scale bars = 100, 50 µm. (F) Immunofluorescence staining of Cyclin D1. Scale bars = 100, 50 µm. (G) Immunofluorescence staining of p21. Scale bars = 100, 50 µm.

Following the demonstration of efficacy in subcutaneous models, we further evaluated the therapeutic potential of ICG/siCD24@MSN‐LCD+US in an orthotopic liver cancer model to better recapitulate the clinical tumor. Successful establishment of the orthotopic model was first confirmed via IVIS and ultrasound imaging (Figure S28). IVIS revealed distinct bioluminescence signals localized to the hepatic region, while corresponding grayscale ultrasound showed hypoechoic tumor masses in the liver. Tumor‐bearing mice received different treatments, following the schedule outlined in Figure 8A. After treatment, livers were harvested for photography and measurement. The ICG/siCD24@MSN‐LCD+US group exhibited the smallest tumors (0.13 ± 0.05 cm in maximum tumor size), showing a significant reduction (**p < 0.01) in size compared to other treatment groups, including ICG@MSN‐LCD+US (0.53 ± 0.11 cm), siCD24@MSN‐LCD+US (0.78 ± 0.16 cm), and ICG/siCD24@MSN‐LCD (0.58 ± 0.15 cm) groups (Figure 8B,C).

To further elucidate the antitumor efficacy, histological analyses were performed on tumor tissues to evaluate the therapeutic effect of the ICG/siCD24@MSN‐LCD+US group (Figure 8D–G). The results demonstrated that the ICG/siCD24@MSN‐LCD+US treatment induced the most substantial tumor necrosis and significantly suppressed tumor proliferation (***p < 0.001) (Figure S29) and cell cycle. This was corroborated by decreased expression of Cyclin D1 (*p < 0.05, ICG/siCD24@MSN‐LCD+US vs. ICG@MSN‐LCD+US; ***p < 0.001, ICG/siCD24@MSN‐LCD+US vs. siCD24@MSN‐LCD+US; **p < 0.01, ICG/siCD24@MSN‐LCD+US vs. ICG/siCD24@MSN‐LCD) (Figure S30) and increased expression of p21 (***p < 0.001, ICG/siCD24@MSN‐LCD+US vs. ICG@MSN‐LCD+US, siCD24@MSN‐LCD+US, ICG/siCD24@MSN‐LCD) (Figure S31), collectively confirming the superior effectiveness of the combined therapy over individual monotherapies. Furthermore, body weight of mice showed no obvious change across the different treatment groups throughout the study period (Figure S32), confirming the safety profile of the treatment.

## 4 Conclusion

In summary, we developed a multifunctional theranostic nanoplatform (ICG/siCD24@MSN‐LCD) with enhanced cellular uptake for effective sono‐gene therapy. Under ultrasound irradiation, ICG/siCD24@MSN‐LCD simultaneously executes dual therapeutic modalities: ICG‐mediated SDT generates cytotoxic ROS and siCD24‐mediated CD24 silencing, both of which synergistically activate the p53 signaling axis. Moreover, the nanoplatform serves as a biocompatible ultrasound imaging agent for real‐time diagnostic monitoring with negligible systemic toxicity. Both in vitro and in vivo studies consistently demonstrate the superior therapeutic effect of this sono‐gene combinatorial approach compared with monotherapies. Despite the above satisfactory performance, this study still faces certain limitations toward further optimization and clinical translation. The multi‐step fabrication involving MSN preparation, ICG/siCD24 co‐loading, and LCD modification complicates large‐scale production and batch‐to‐batch quality control. Additionally, further evaluation in higher‐order animal models, long‐term in vivo metabolism assessment, and improvement of deep tumor penetration are still required to facilitate clinical translation. Even so, this work still provides a feasible strategy that integrates sono‐gene therapy with ultrasound imaging for precise HCC treatment. It not only offers a versatile nanoplatform for image‐guided tumor intervention, but also provides valuable insights into the development of personalized cancer nanomedicine.

## Author Contributions

Y.D.Z. designed the study, supervised the project, and drafted the original manuscript. Reviewing and editing were completed by L.G., D.D.S., X.S., and M.M.S. Methodology was developed by S.N., S.T.H., and X.X.W. Investigation was carried out by R.L., Y.Y.F., and S.Y.L. Conceptualization and supervision were provided by J.L. Funding acquisition was obtained by J.L. and Y.D.Z.

## Conflicts of Interest

The authors declare no conflicts of interest.

## Supporting information




**Supporting File**: advs76922‐sup‐0001‐SuppMat.pdf

## Data Availability

The data that support the findings of this study are available from the corresponding author upon reasonable request.
