# Supplementary material for: Synergistic p53 Pathway Activation Through Sono‐Gene Therapy Induced by Ultrasound‐Triggered Theranostic Mesoporous Nanoparticles
Source: Adv Sci (Weinh). 2026 Aug 3;13(60):e76922. doi: 10.1002/advs.76922 (PMC13430928; doi:10.1002/advs.76922)
Supplement: Supplementary file 1 [file ADVS-13-e76922-s001.pdf]

Synergistic p53 Pathway Activation through Sono-Gene Therapy Induced by Ultrasound-Triggered Theranostic Mesoporous Nanoparticles

Yading Zhao, Lu Guo, Dandan Shi, Xiao Sun, Mengmeng Shang, Song Ning, Shuting Huang, Xiaoxuan Wang, Rui Liu, Yuye Fu, Suyun Li, Jie Li*

*Corresponding authors: Tel/fax: +86-531-82166101. Email address: jieli@email.sdu.edu.cn.

Department of Ultrasound, Qilu Hospital of Shandong University, Jinan, Shandong 250012, China

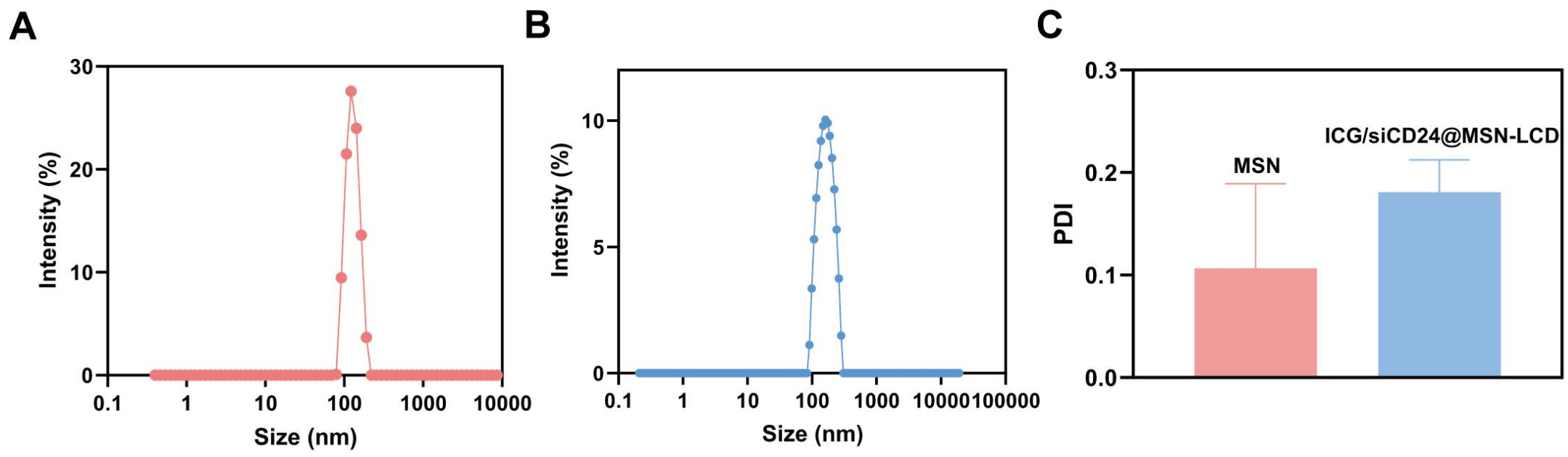

Figure S1. (A) Size distribution of MSN. **(B)** Size distribution of ICG/siCD24@MSN-LCD. **(C)** PDI of MSN and ICG/siCD24@MSN-LCD.

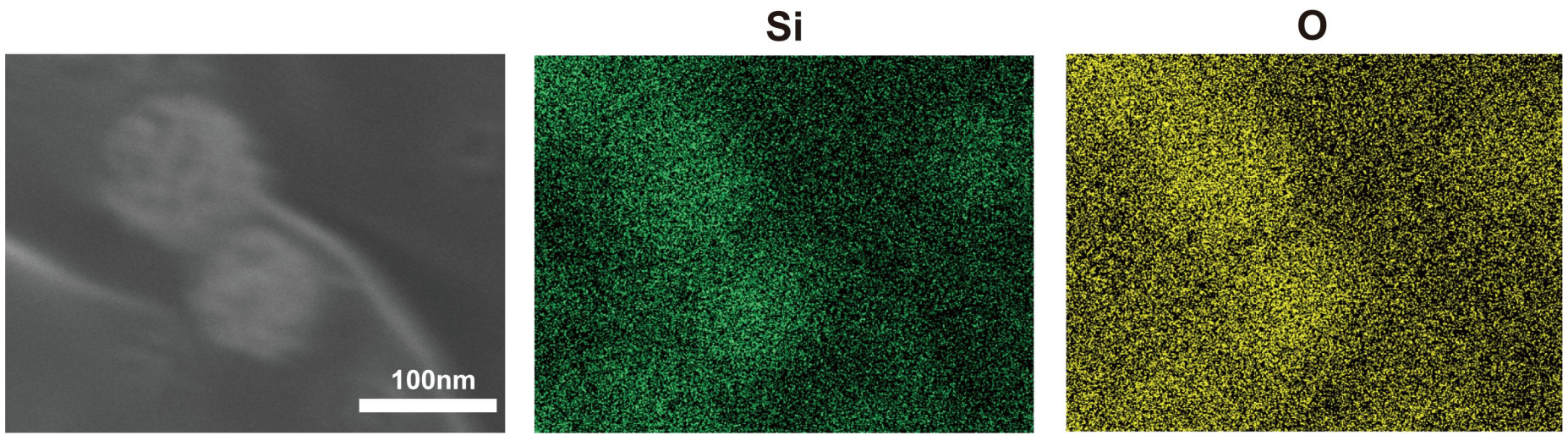

Figure S2. EDS analysis of MSN: Si (green), O (yellow). Scale bar = 100 nm.

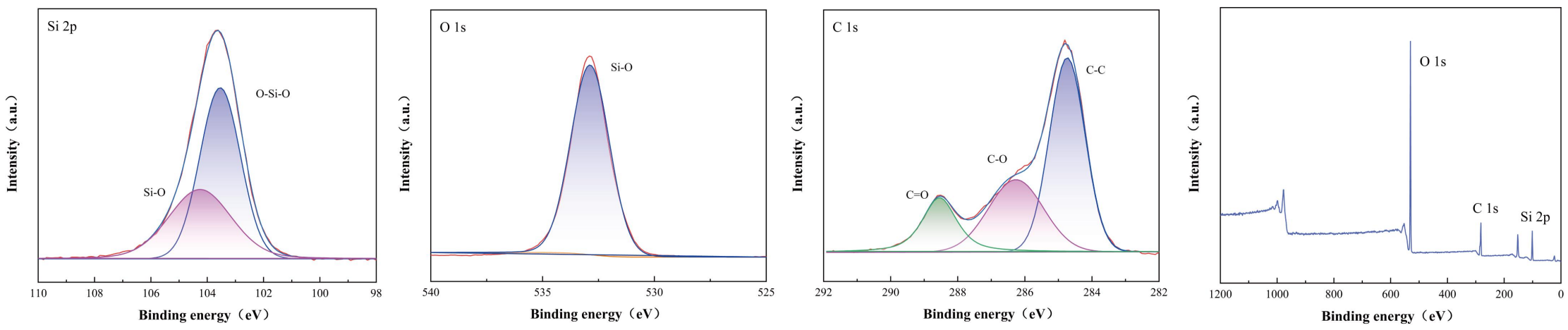

Figure S3. XPS spectra of MSN, including the high-resolution spectrum of Si 2p, O 1s, and C 1s and survey spectrum.

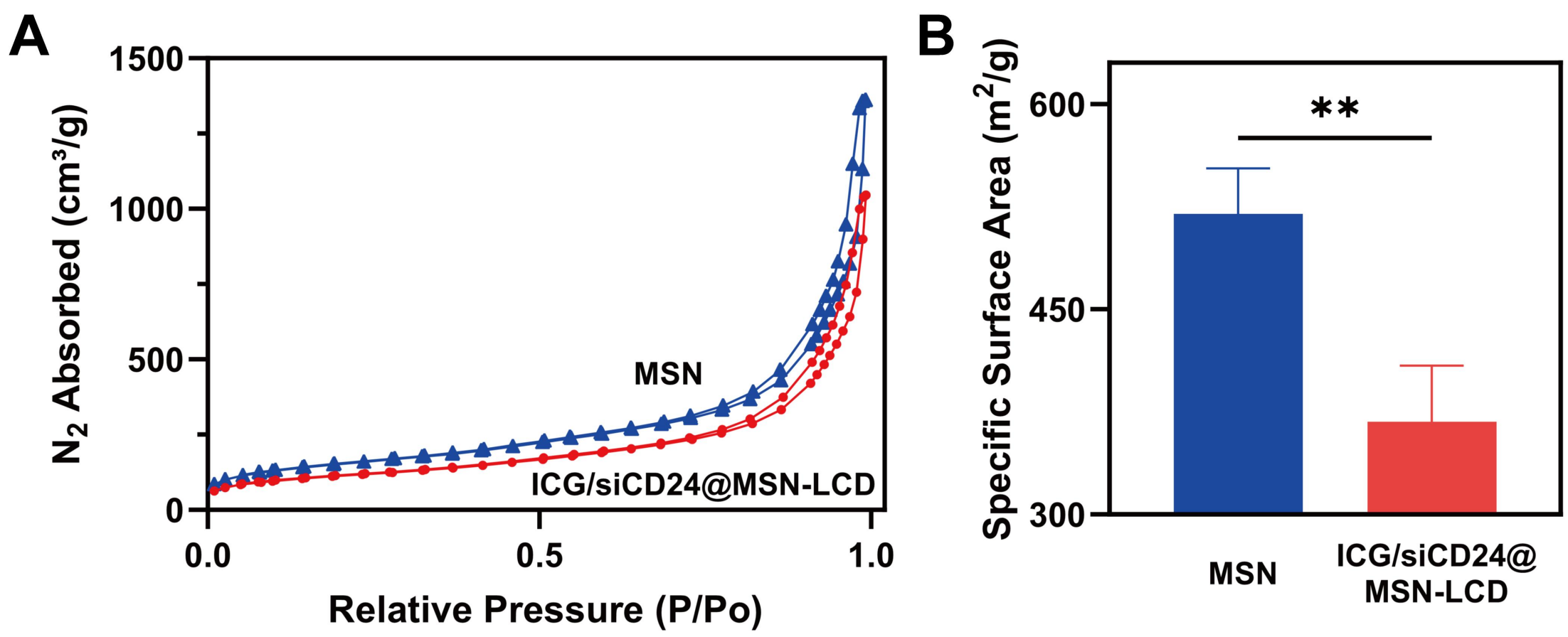

Figure S4. (A) BET result of MSN and ICG/siCD24@MSN-LCD. **(B)** Specific surface area of MSN and ICG/siCD24@MSN-LCD.

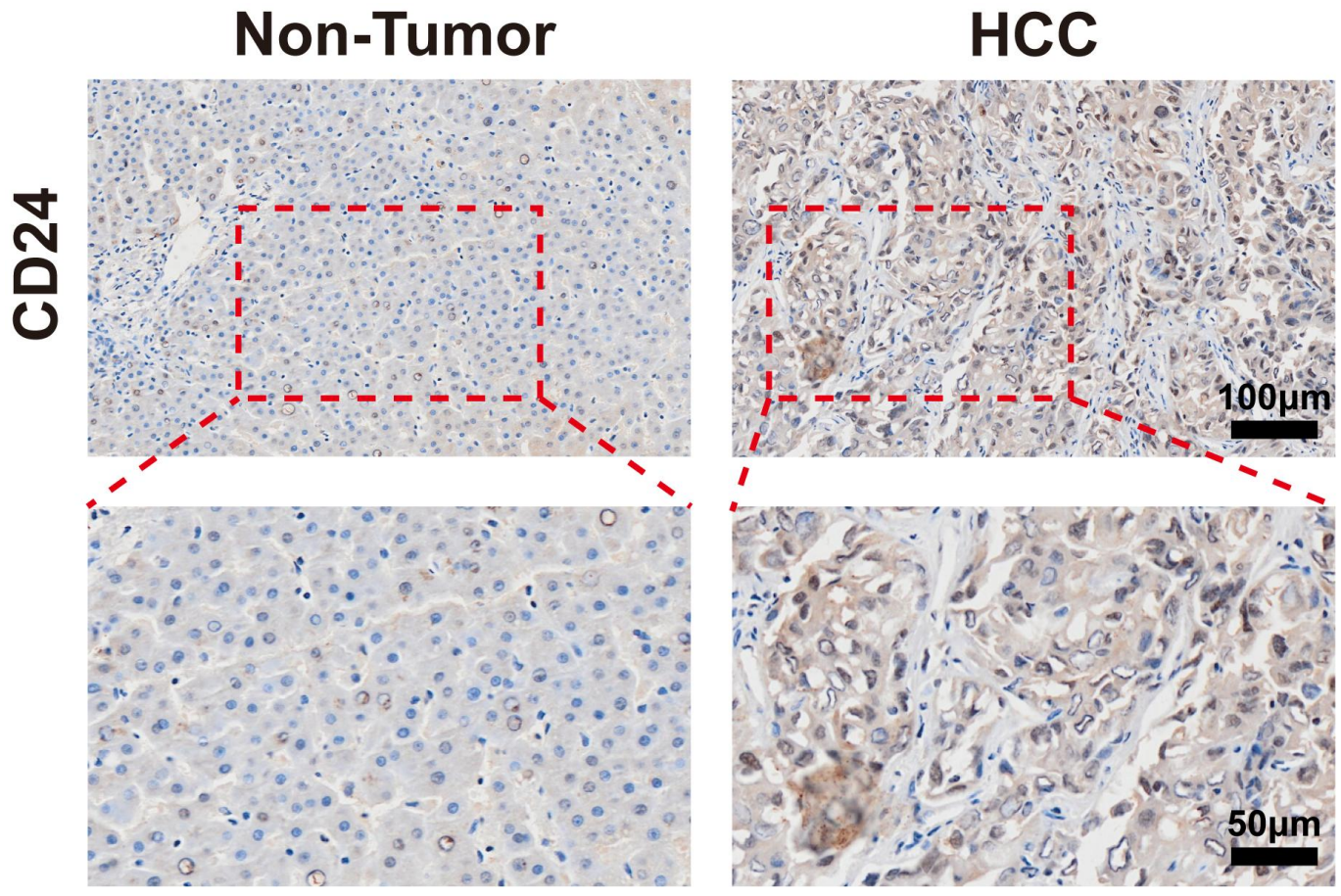

Figure S5. Immunohistochemical staining of CD24. Scale bars = 100 µm, 50µm.

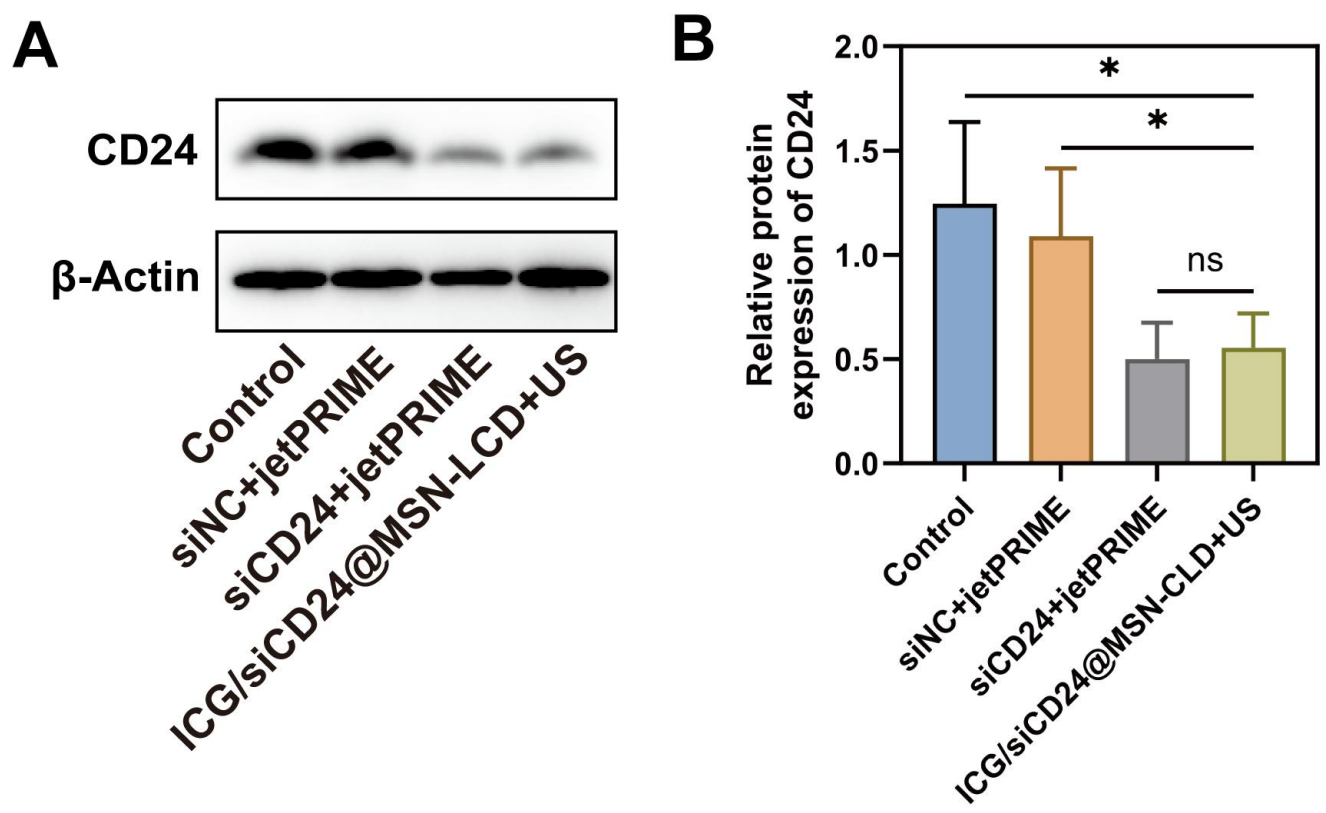

Figure S6. (A) CD24 silencing under different treatment conditions. **(B)** Quantitative analysis of CD24 expression from A.

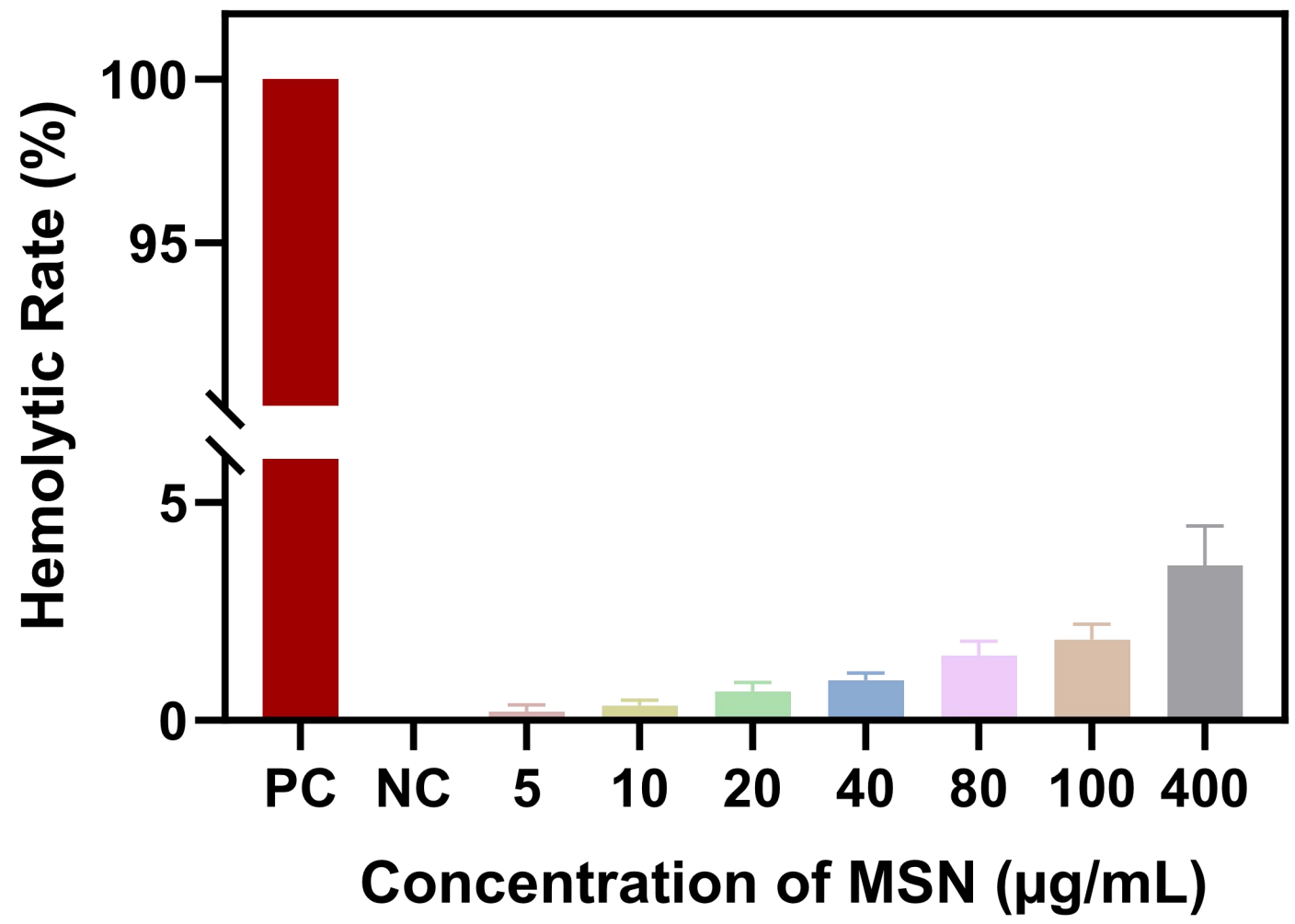

Figure S7. The hemolysis with different doses of MSN.

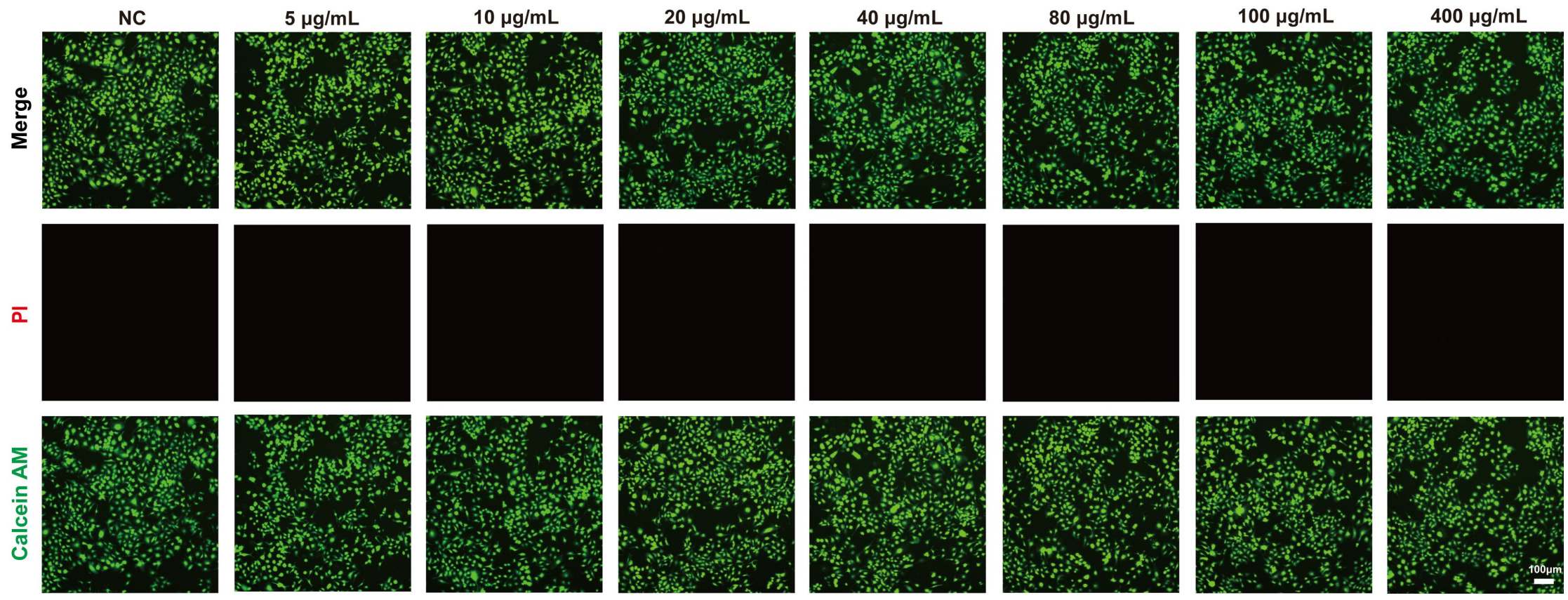

Figure S8. The Live and Dead staining of Huh7 cells with different doses of MSN. Scale bar = 100 μm.

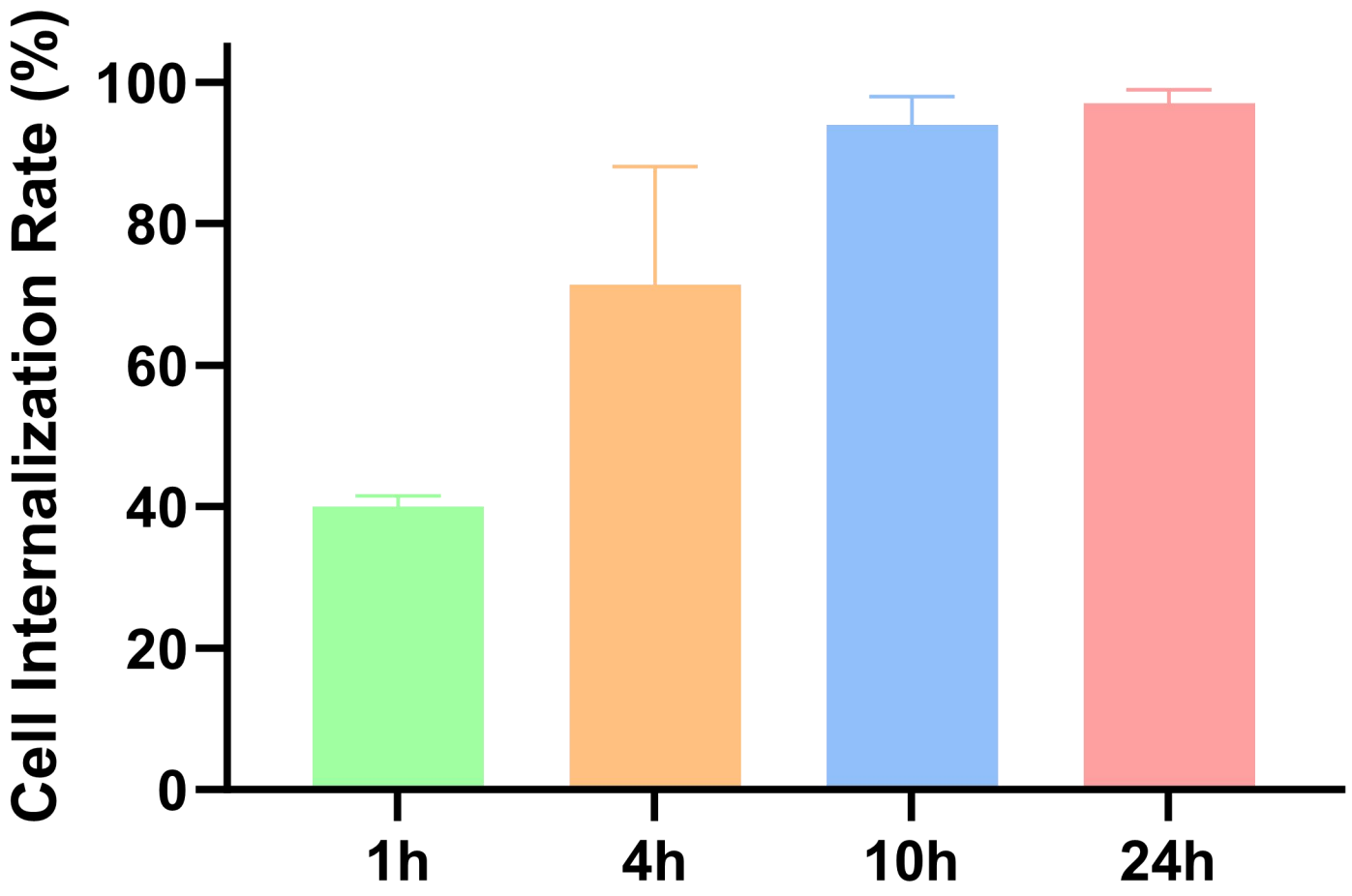

Figure S9. Cellular uptake of nanoparticles at different incubation times, quantified by FCM.

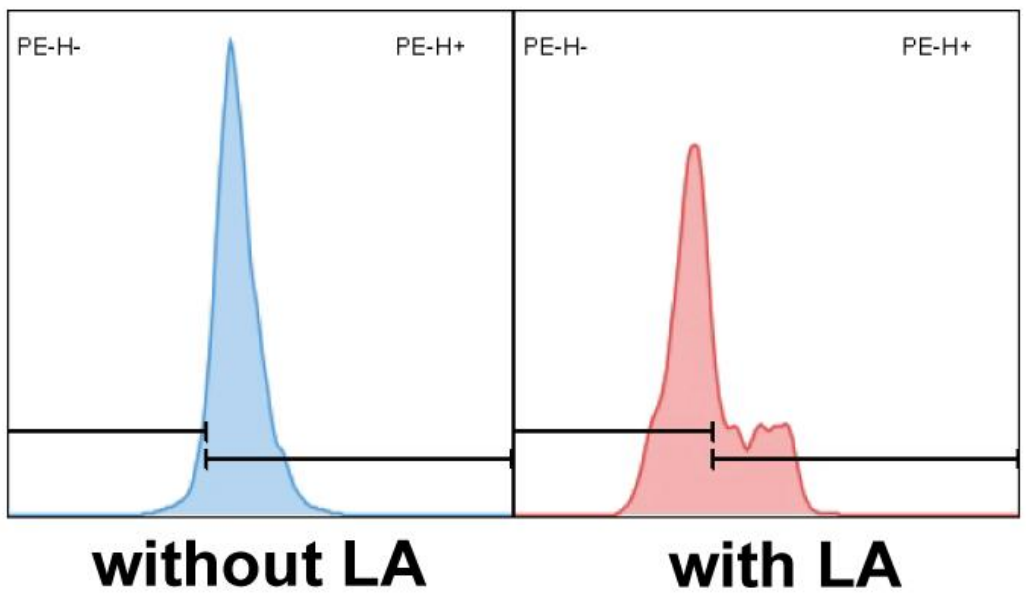

Figure S10. FCM analysis from cellular adhesion of ICG/siCD24@MSN-LCD by Huh7 cells with/without LA.

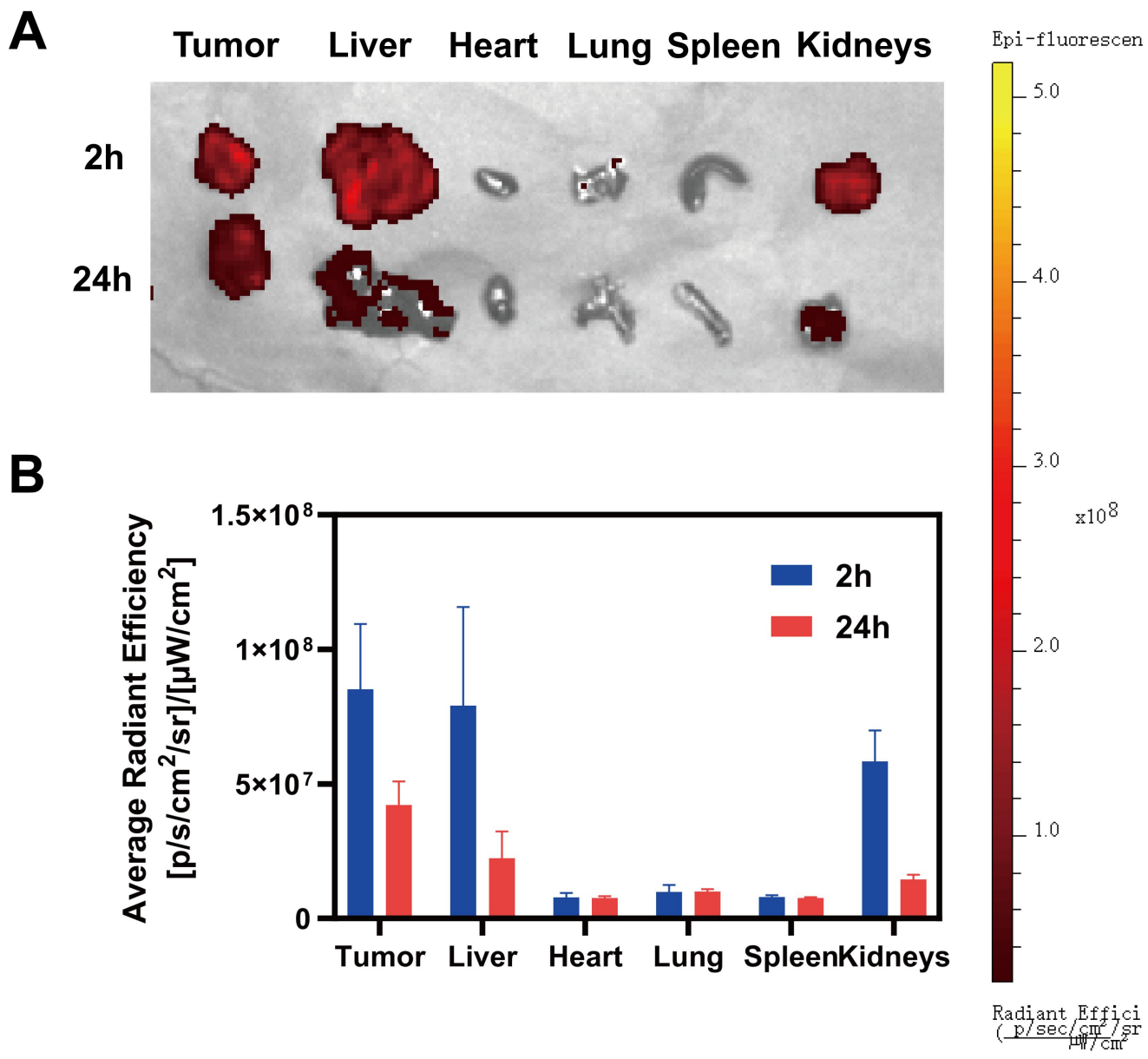

Figure S11. (A) In vivo biodistribution at different time. **(B)** The average radiant efficiency in tumors and major organs measured from A.

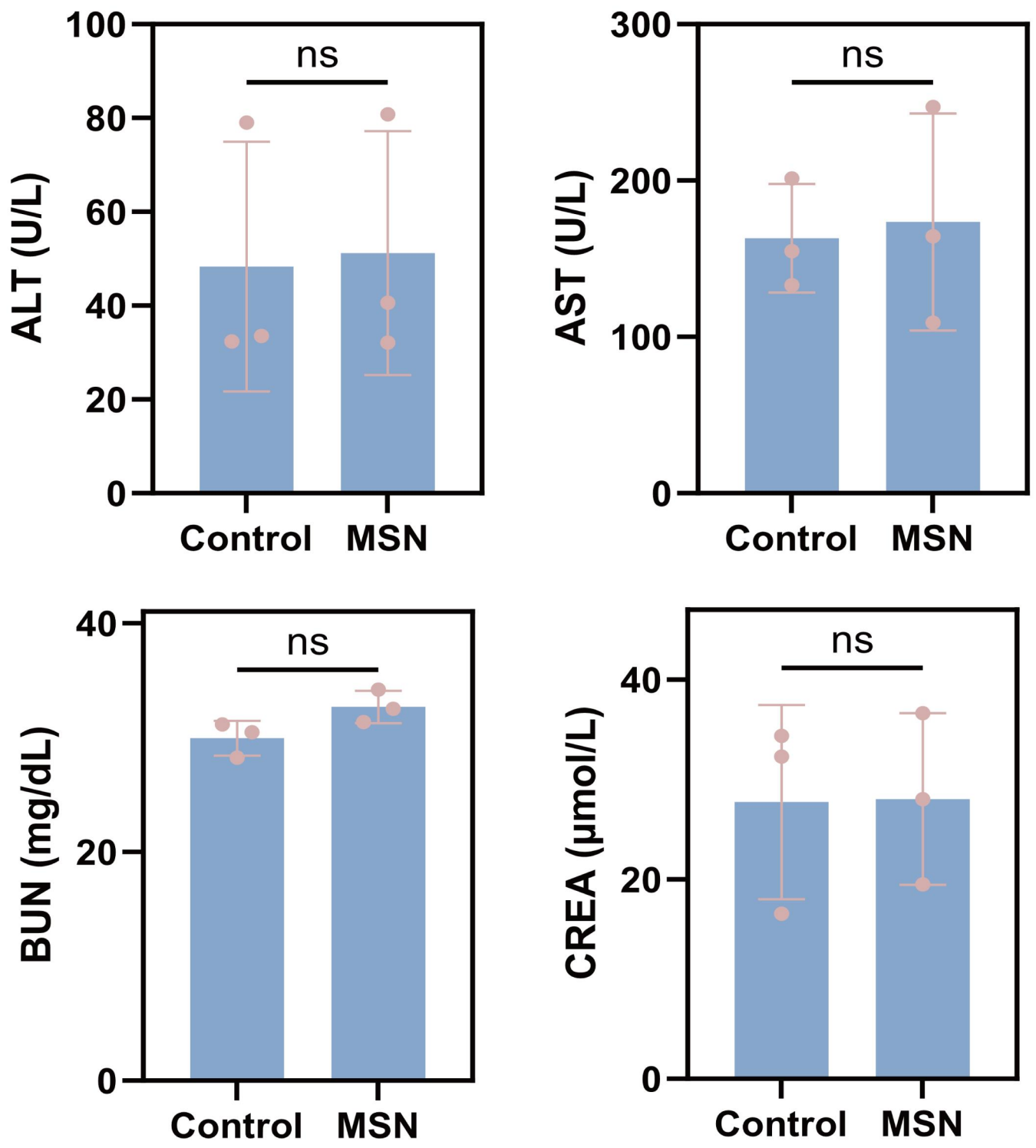

Figure S12. Analyses of serum hepatic and kidney function indicators from mice.

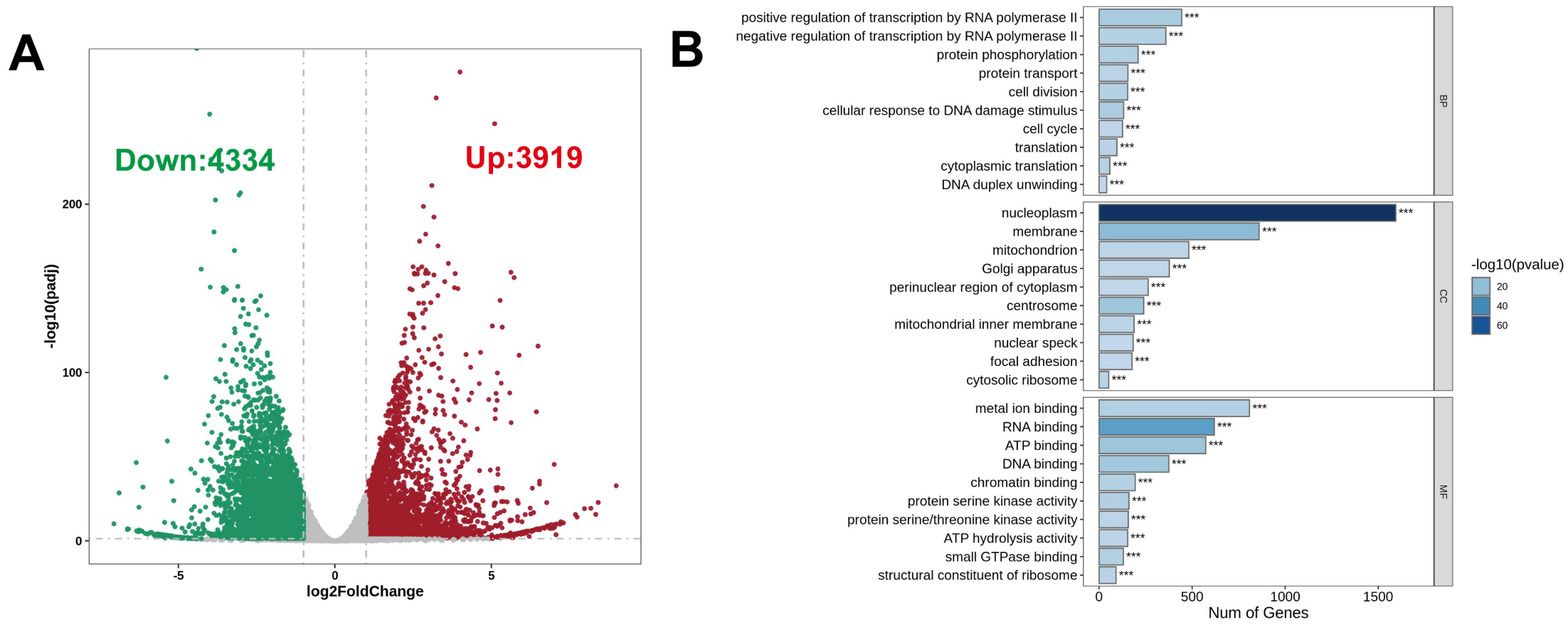

Figure S13. (A) Volcano plot of transcriptome sequencing in Huh7 cells. **(B)** GO enrichment analysis derived from Huh7 cell transcriptome sequencing data.

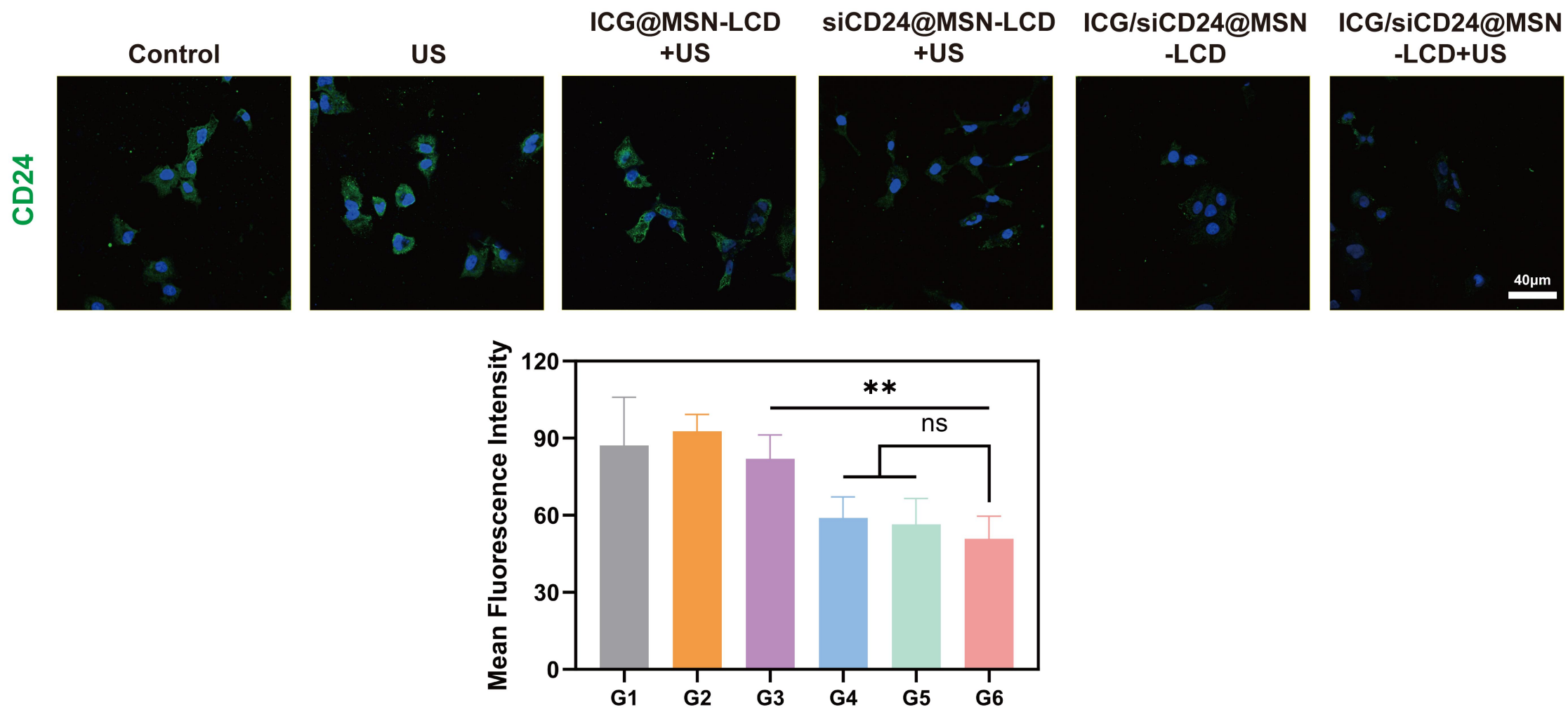

Figure S14. Immunofluorescence image and quantitative analysis of CD24 in Huh7 cells. Scale bar = 40 μm.

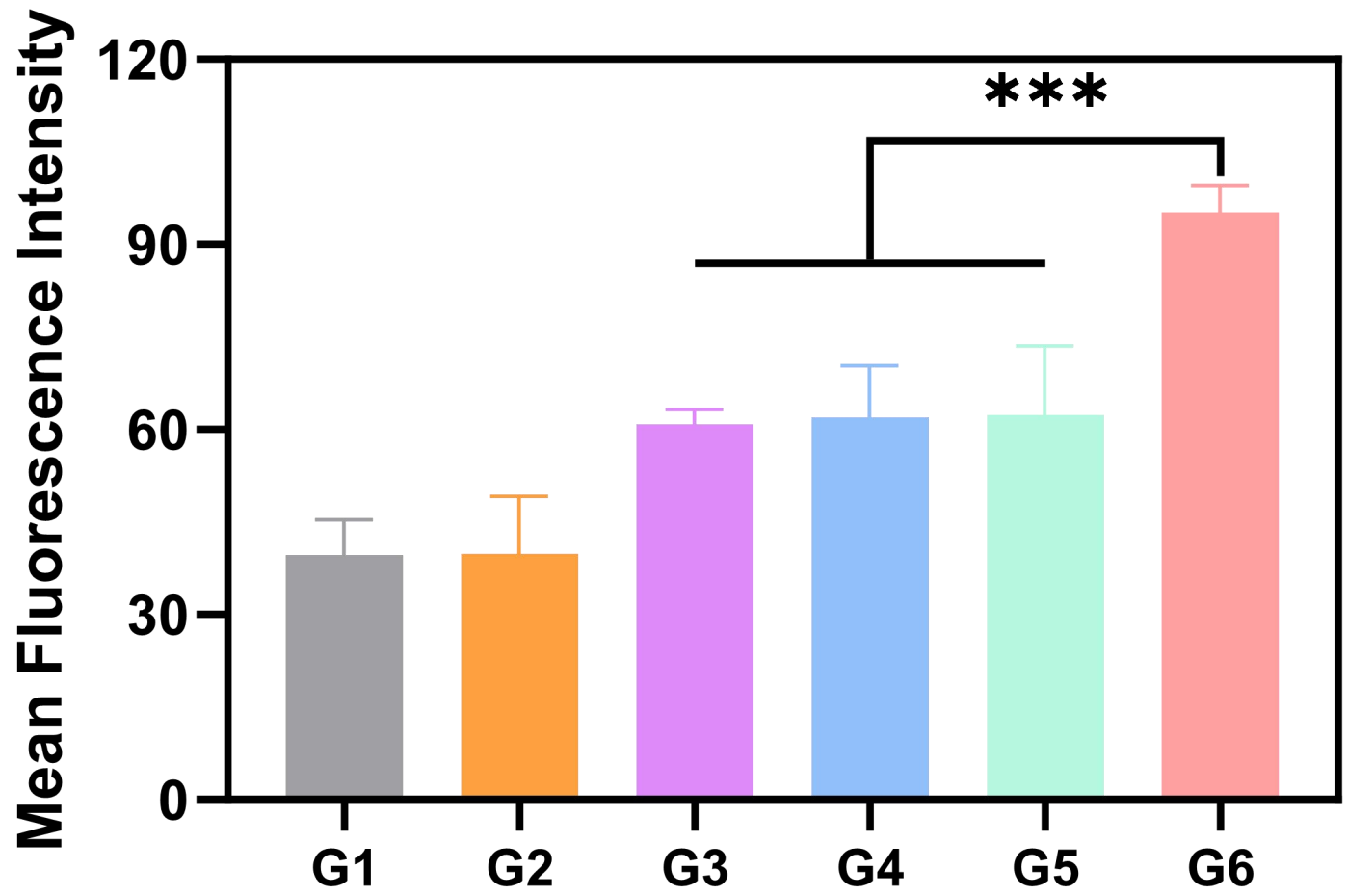

Figure S15. Quantitative analysis of p53 in Huh7 cells measured by mean fluorescence intensity.

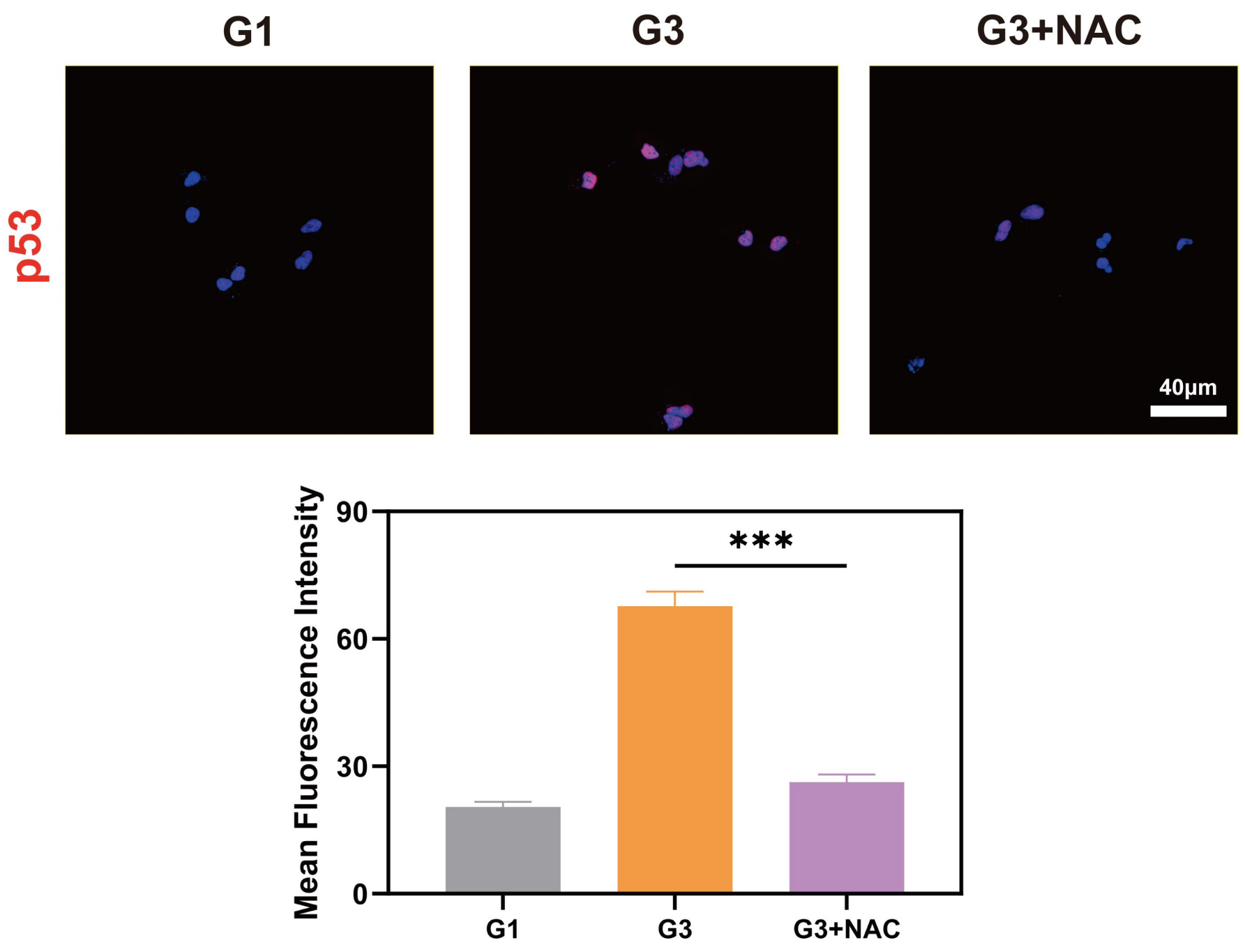

Figure S16. The expression level and quantitative analysis of p53 in Huh7 cells following different treatments. Scale bar = 40 μm.

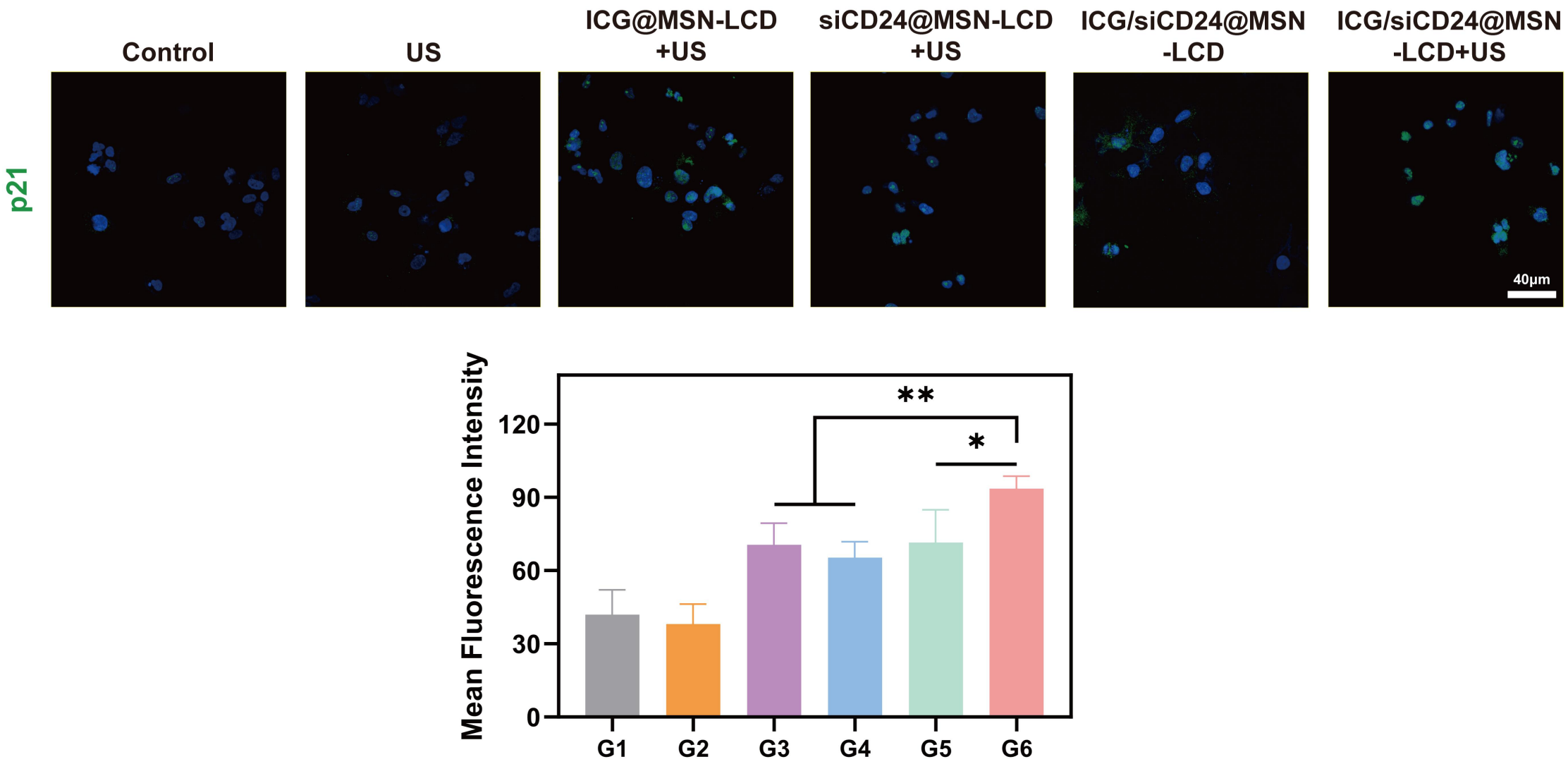

Figure S17. Immunofluorescence image and quantitative analysis of p21 in Huh7 cells. Scale bar = 40 μm.

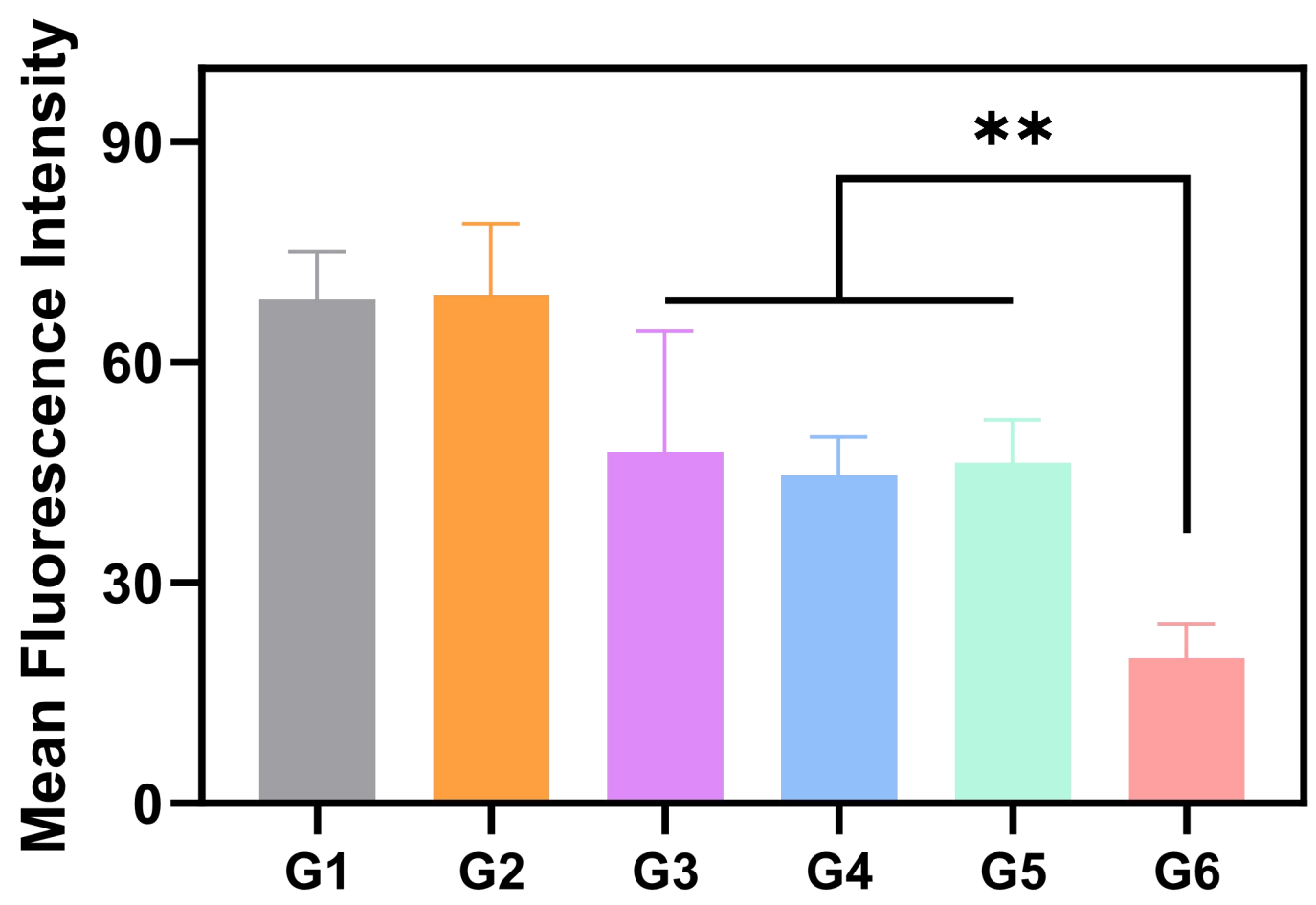

Figure S18. Quantitative analysis of Cyclin D1 in Huh7 cells measured by mean fluorescence intensity.

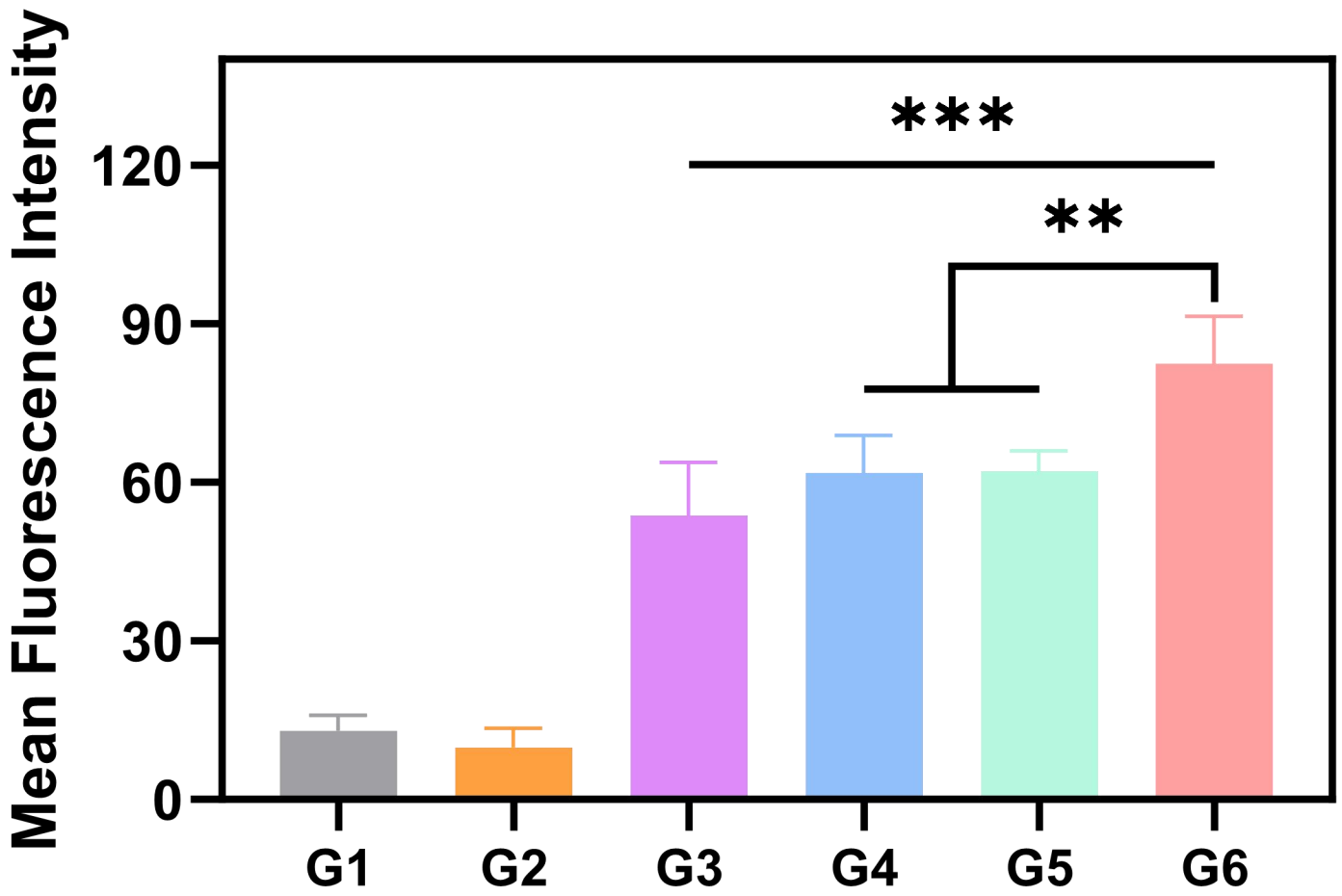

Figure S19. Quantitative analysis of Cleaved caspase-3 in Huh7 cells measured by mean fluorescence intensity.

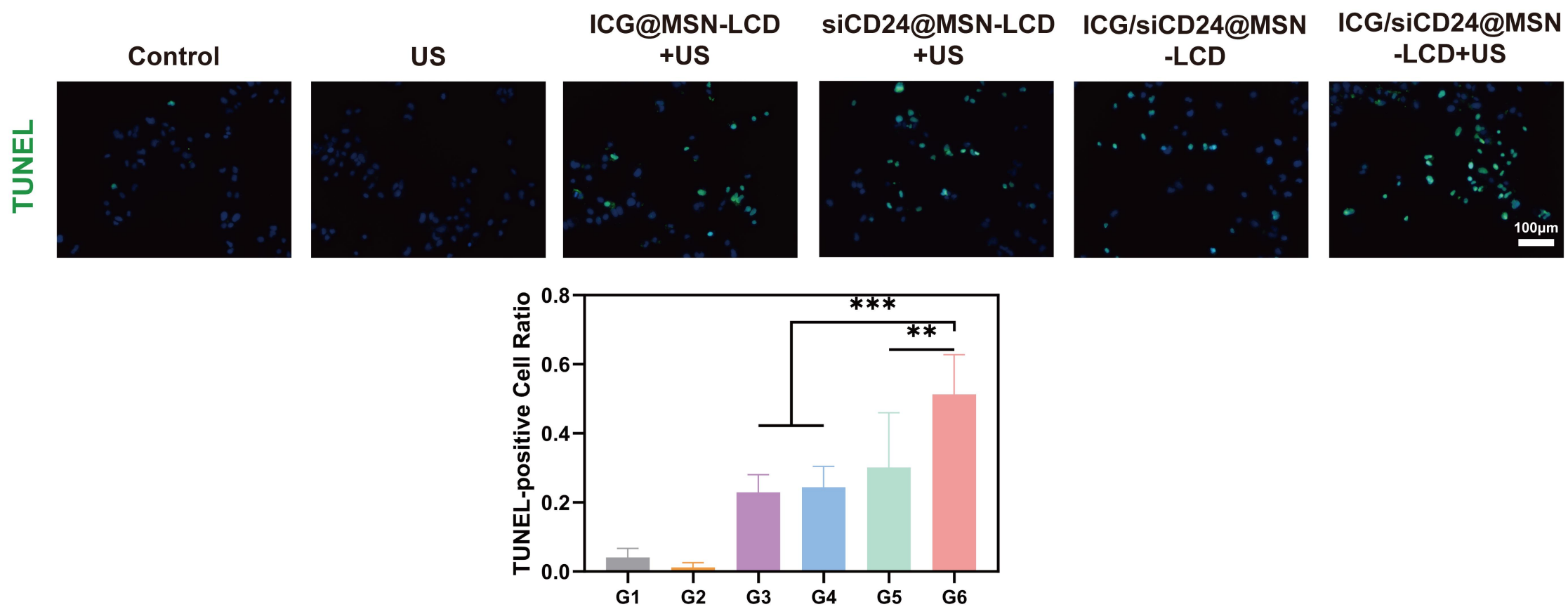

Figure S20. TUNEL staining of Huh7 cells across treatment groups. Scale bar = 100 μm.

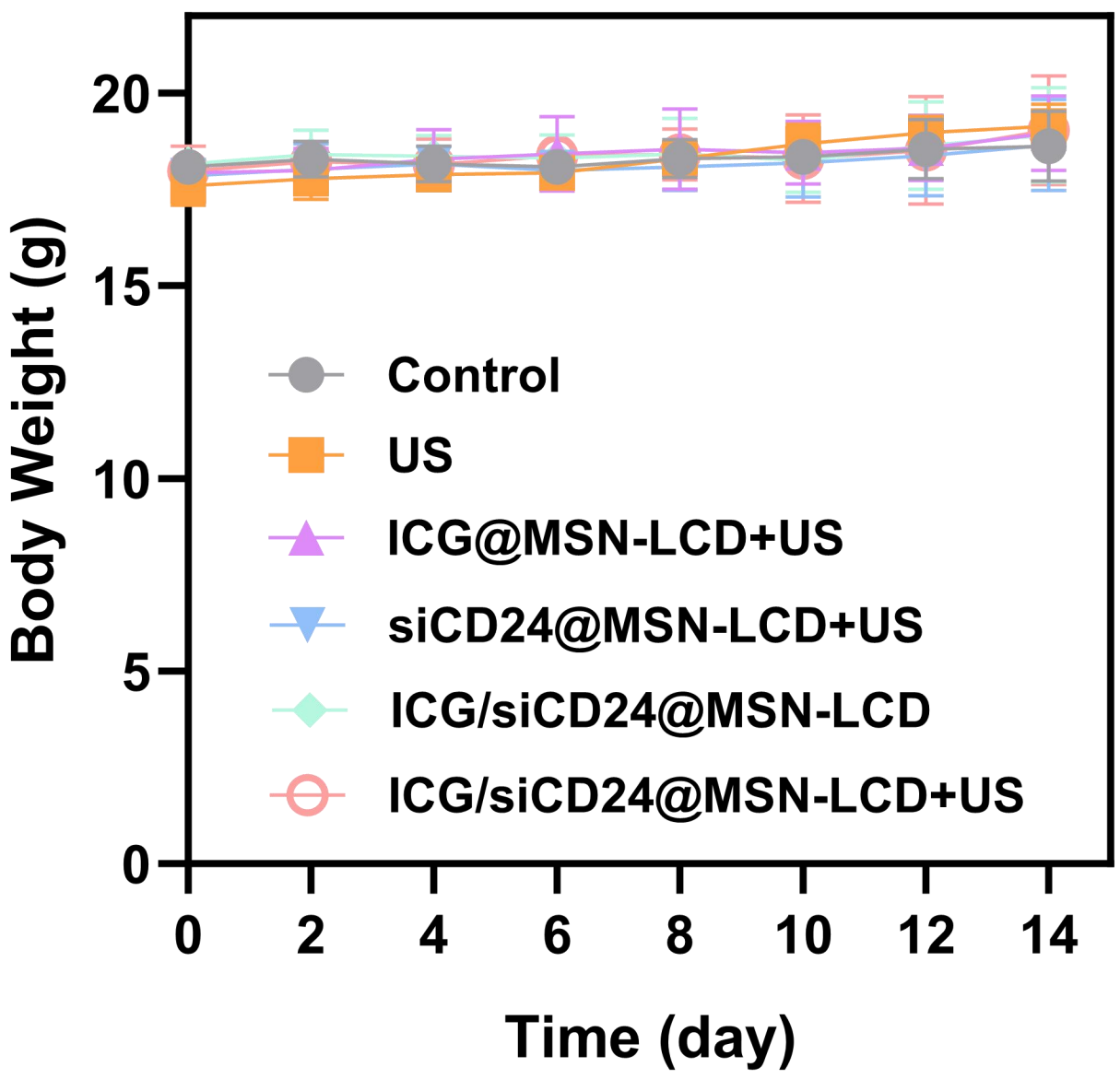

Figure S21. Body weight changes in tumor-bearing nude mice across treatment groups.

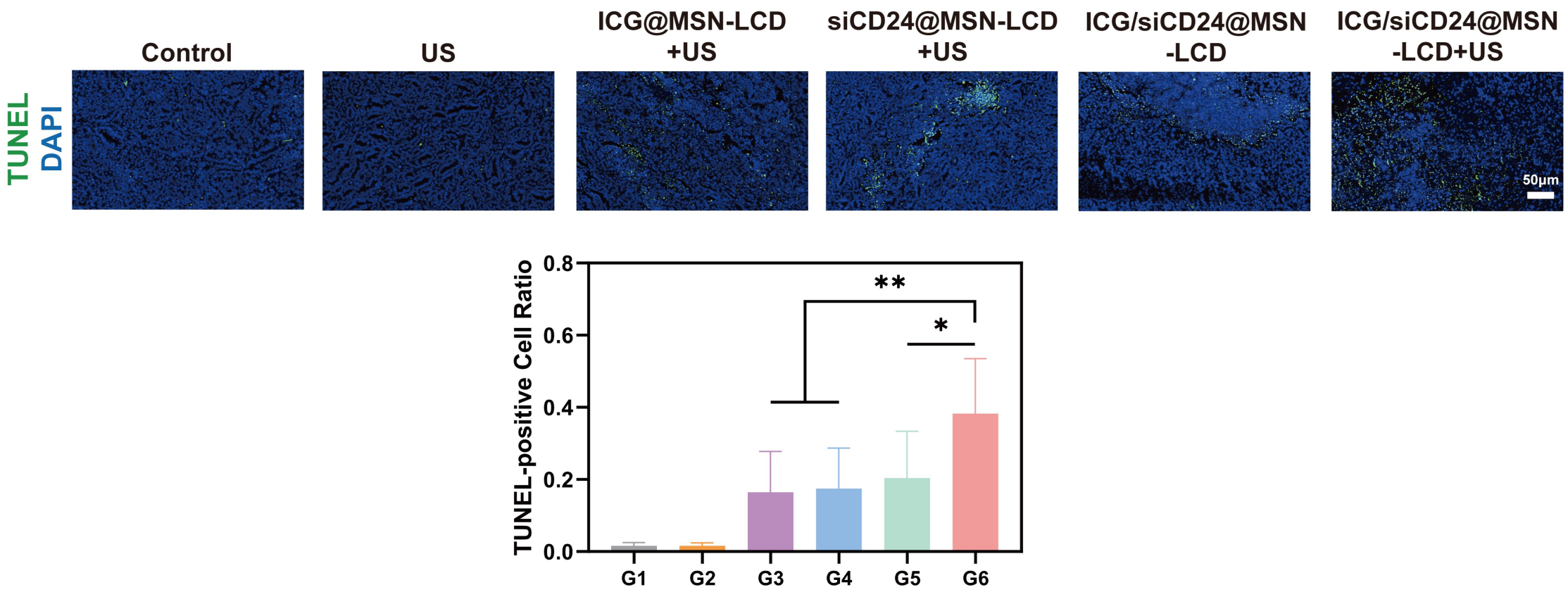

Figure S22. TUNEL staining of tumors in tumor-bearing nude mice across treatment groups. Scale bar = 50 μm.

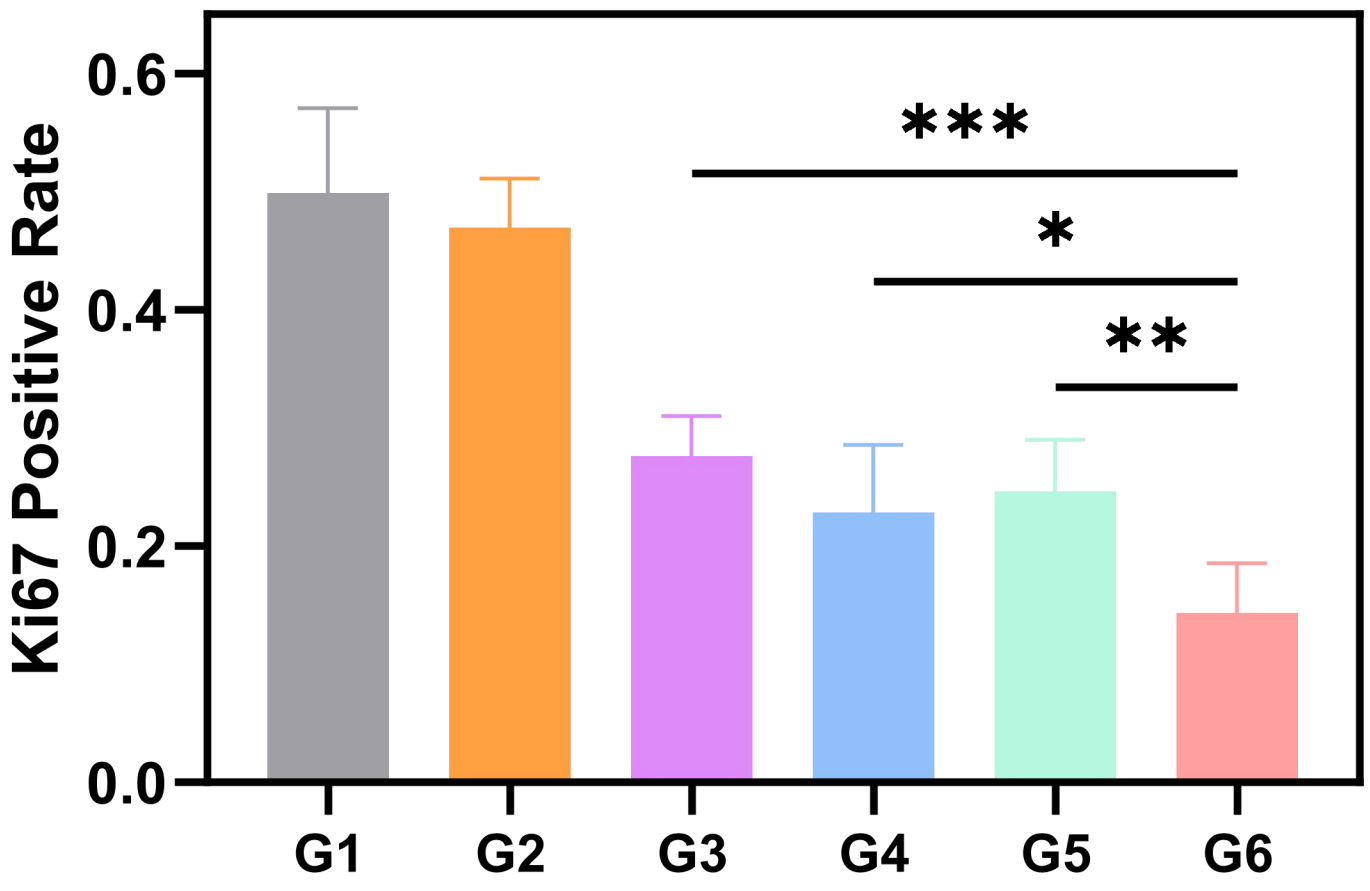

Figure S23. Quantitative analysis of Ki67 in subcutaneous tumors across different treatment.

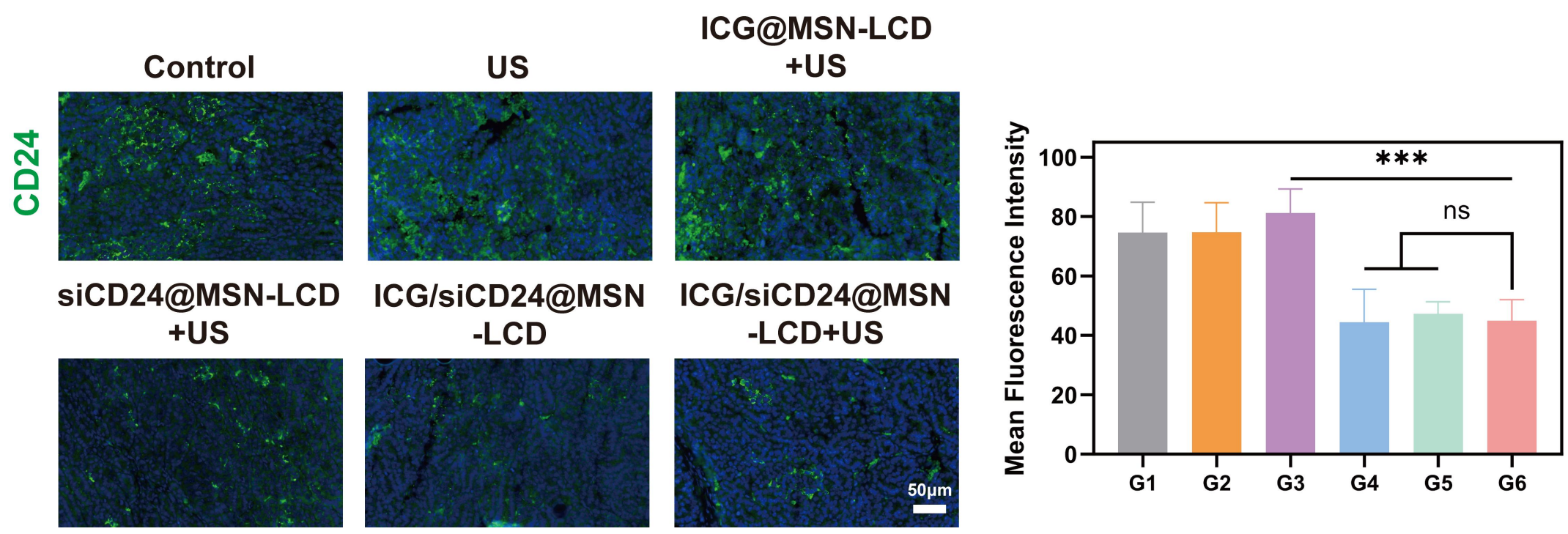

Figure S24. Immunofluorescence image and quantitative analysis of CD24 in subcutaneous tumors across different treatment. Scale bar = 50 μm.

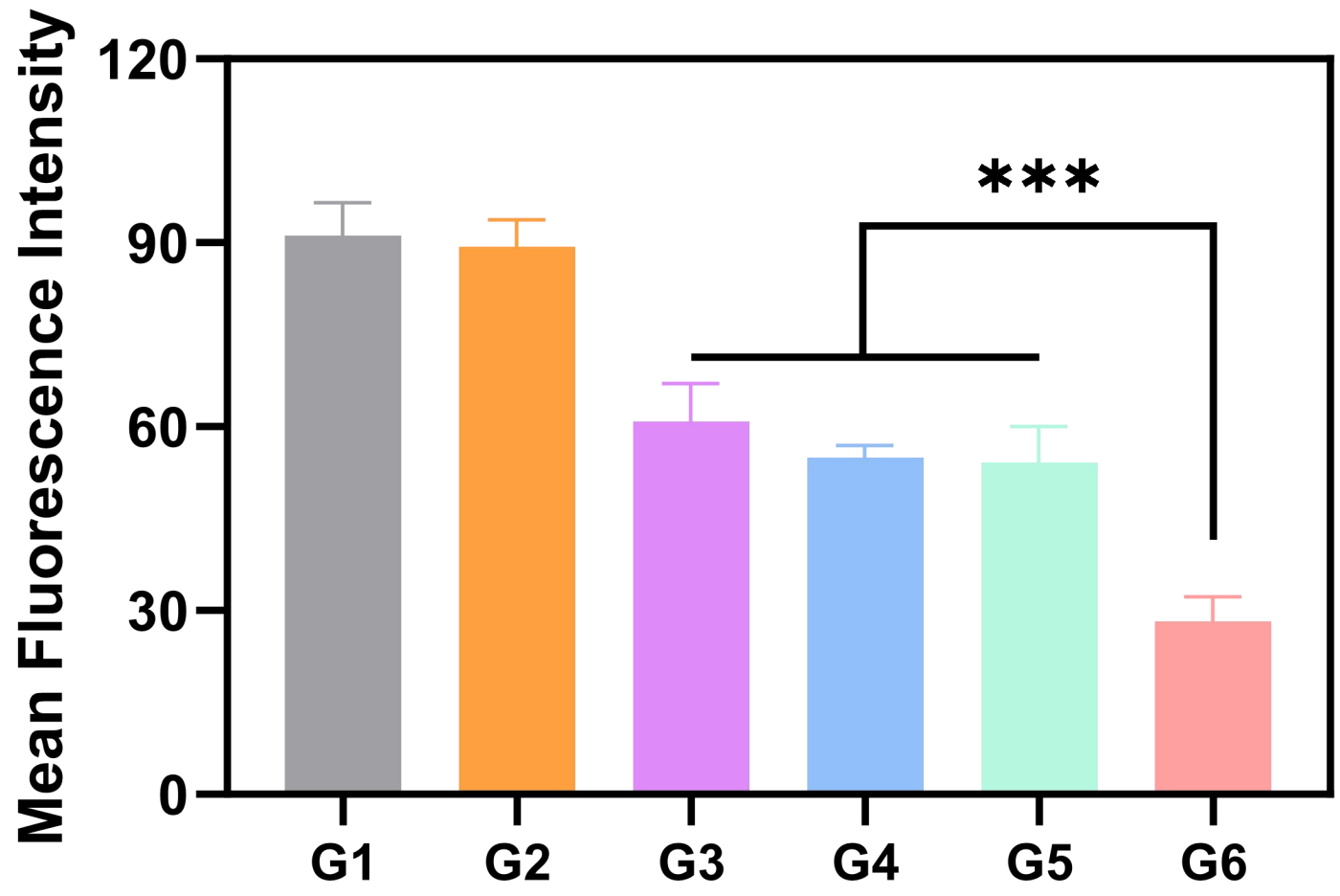

Figure S25. Quantitative analysis of Cyclin D1 in subcutaneous tumors across different treatment.

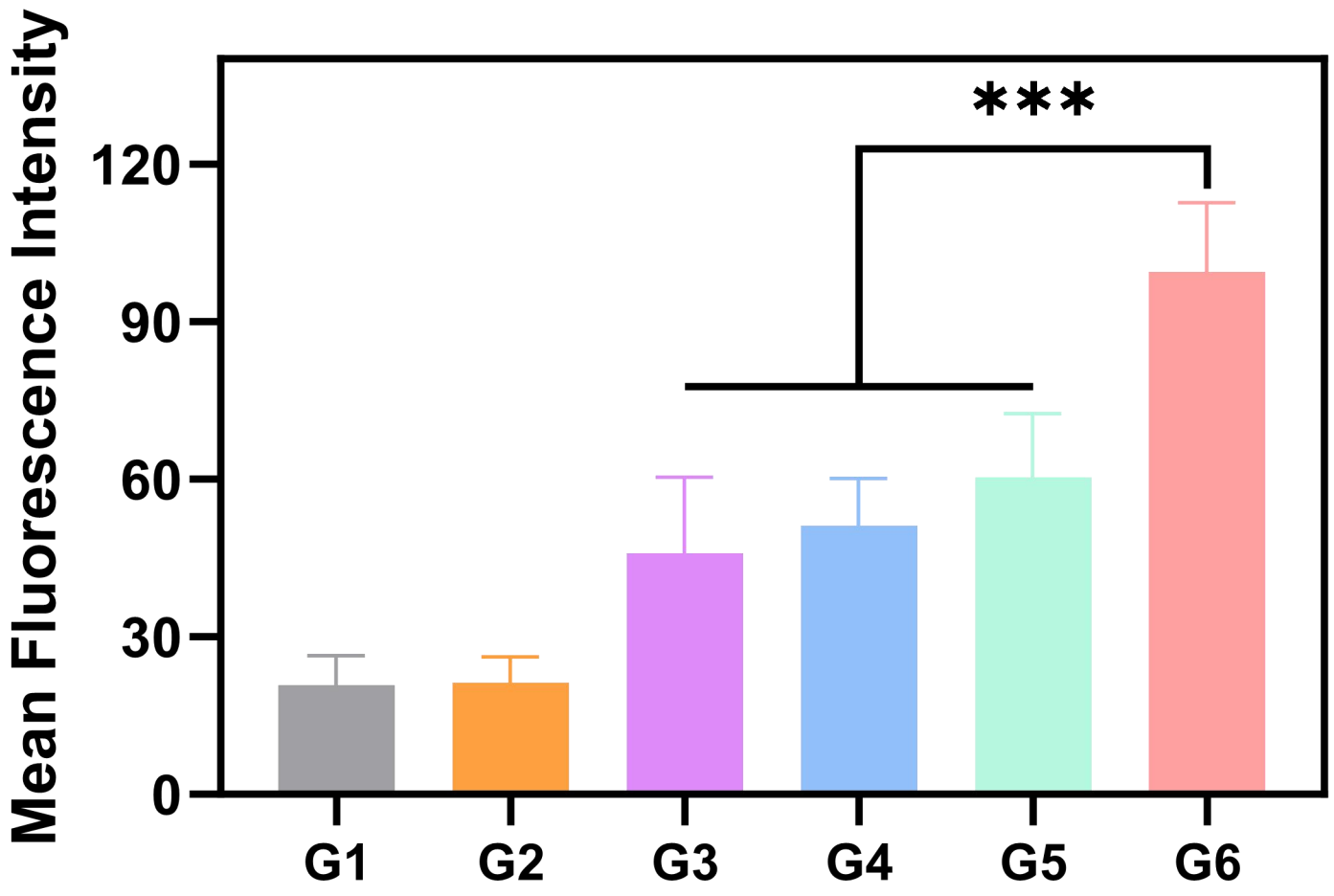

Figure S26. Quantitative analysis of p21 in subcutaneous tumors across different treatment.

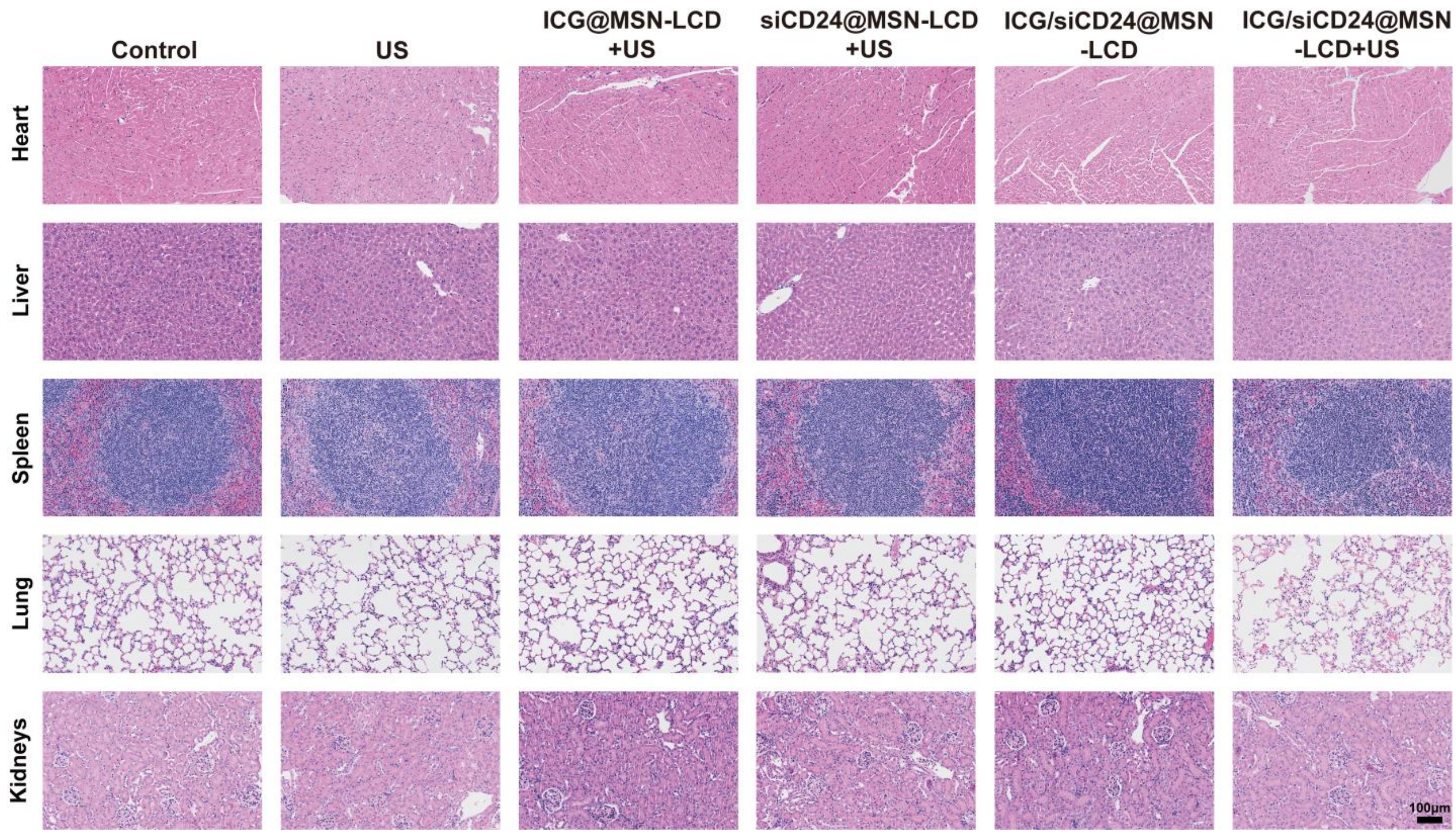

Figure S27. HE staining of major organs in tumor-bearing nude mice across treatment groups. Scale bar = 100 μm.

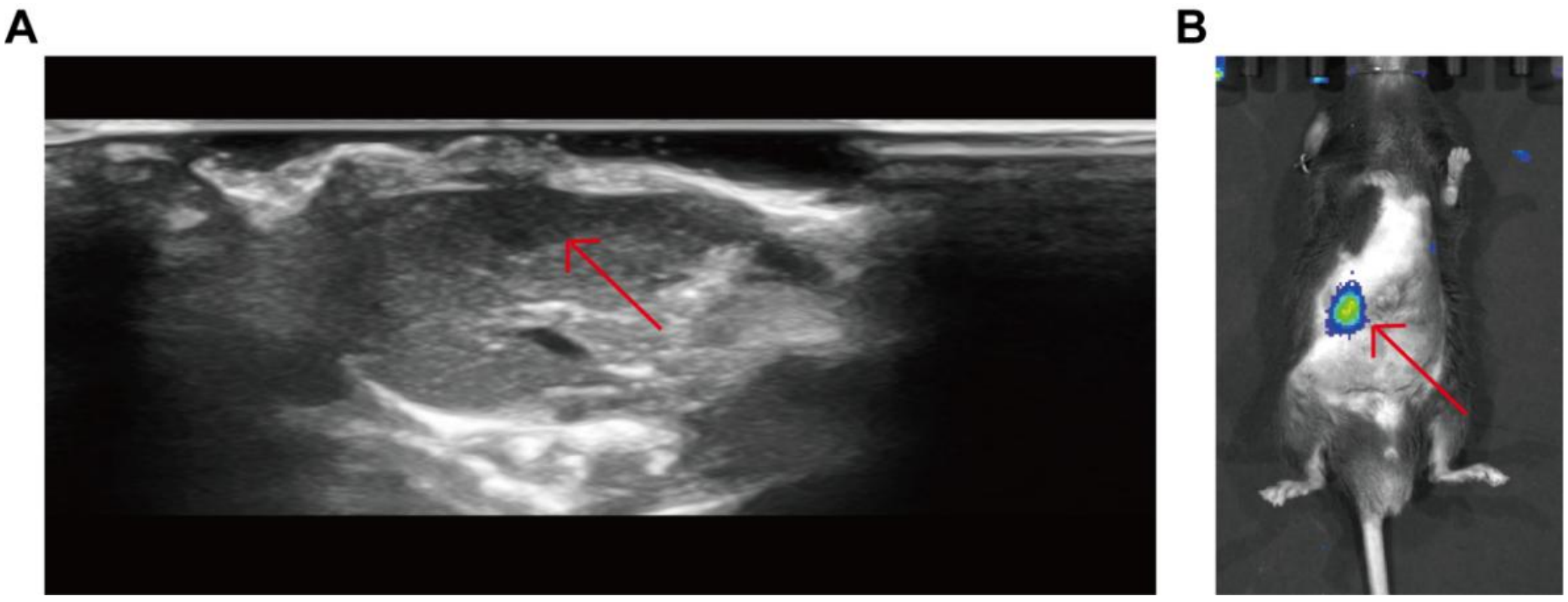

Figure S28. Ultrasound **(A)** and IVIS **(B)** imaging of the orthotopic tumor site in mice. The red arrows indicate the location of the tumor.

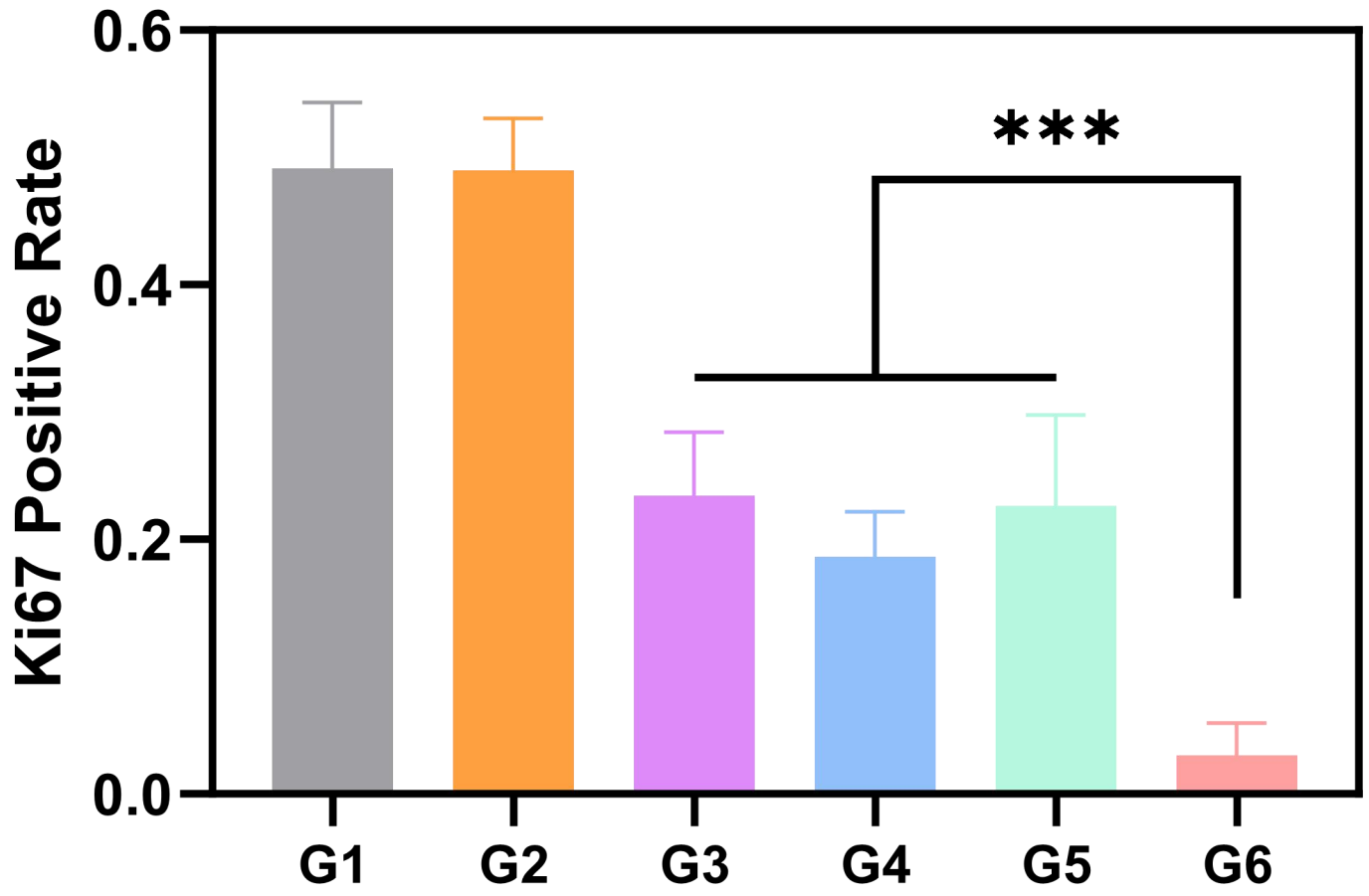

Figure S29. Quantitative analysis of Ki67 of orthotopic tumor tissues.

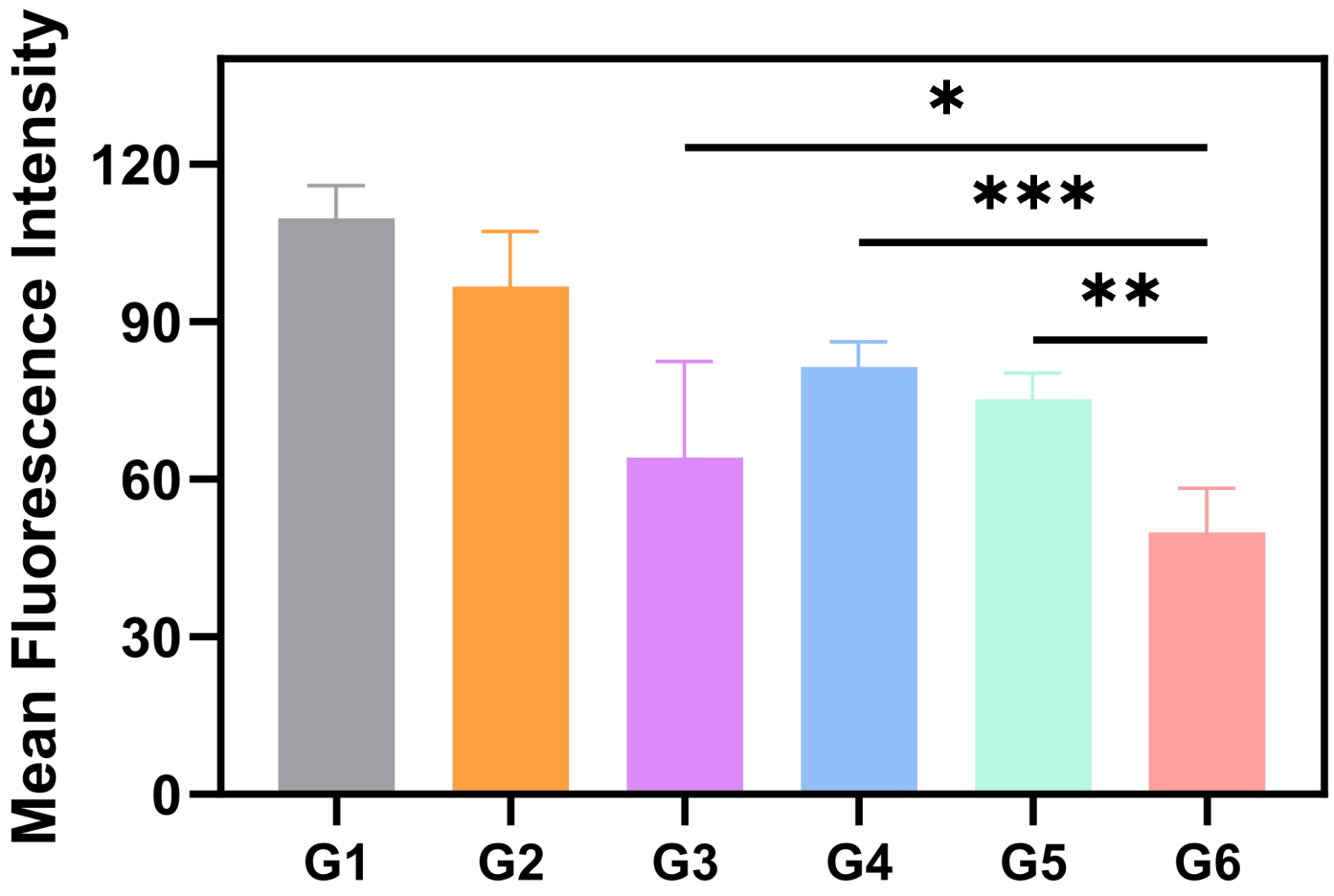

Figure S30. Quantitative analysis of Cyclin D1 of orthotopic tumor tissues.

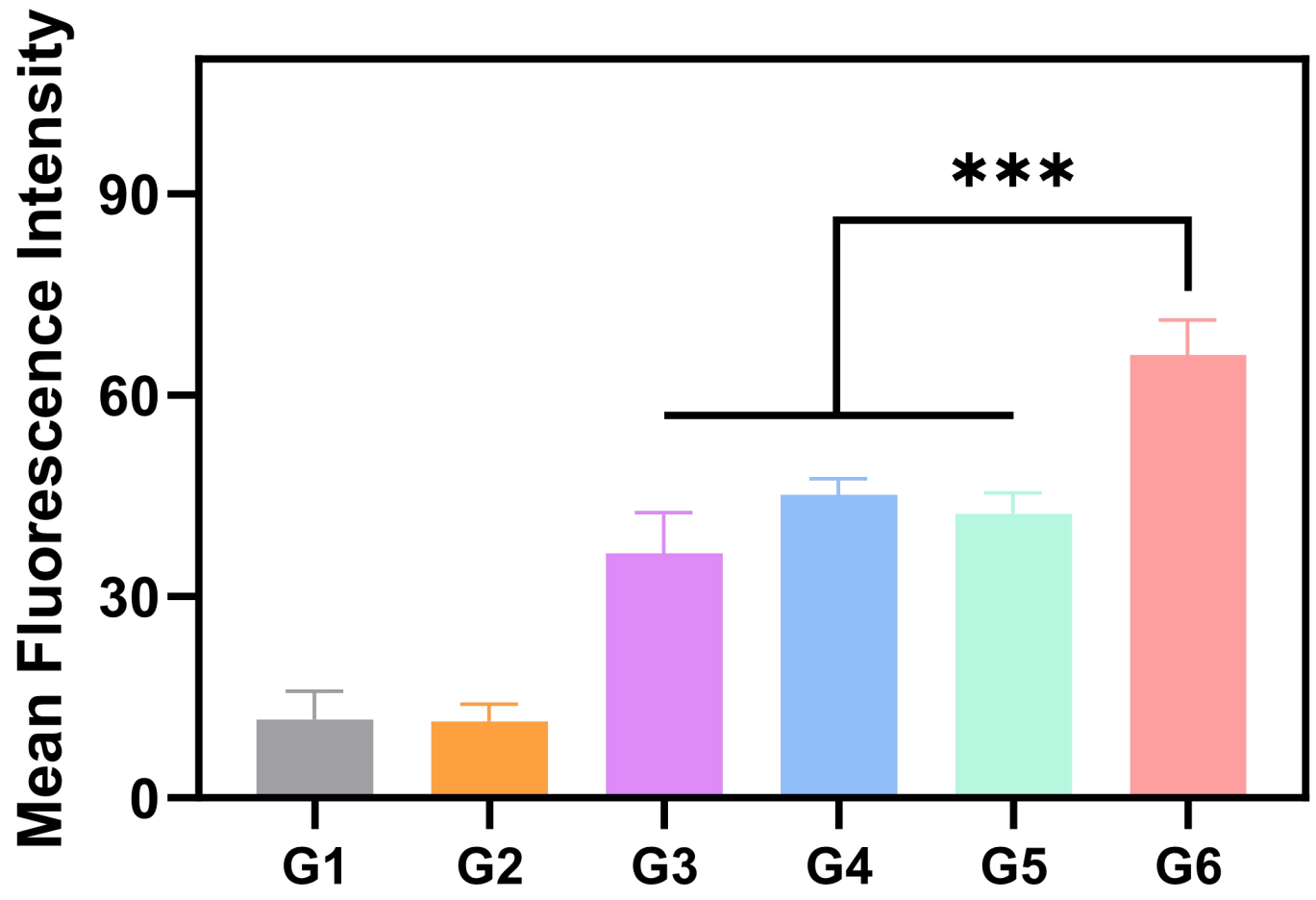

Figure S31. Quantitative analysis of p21 of orthotopic tumor tissues.

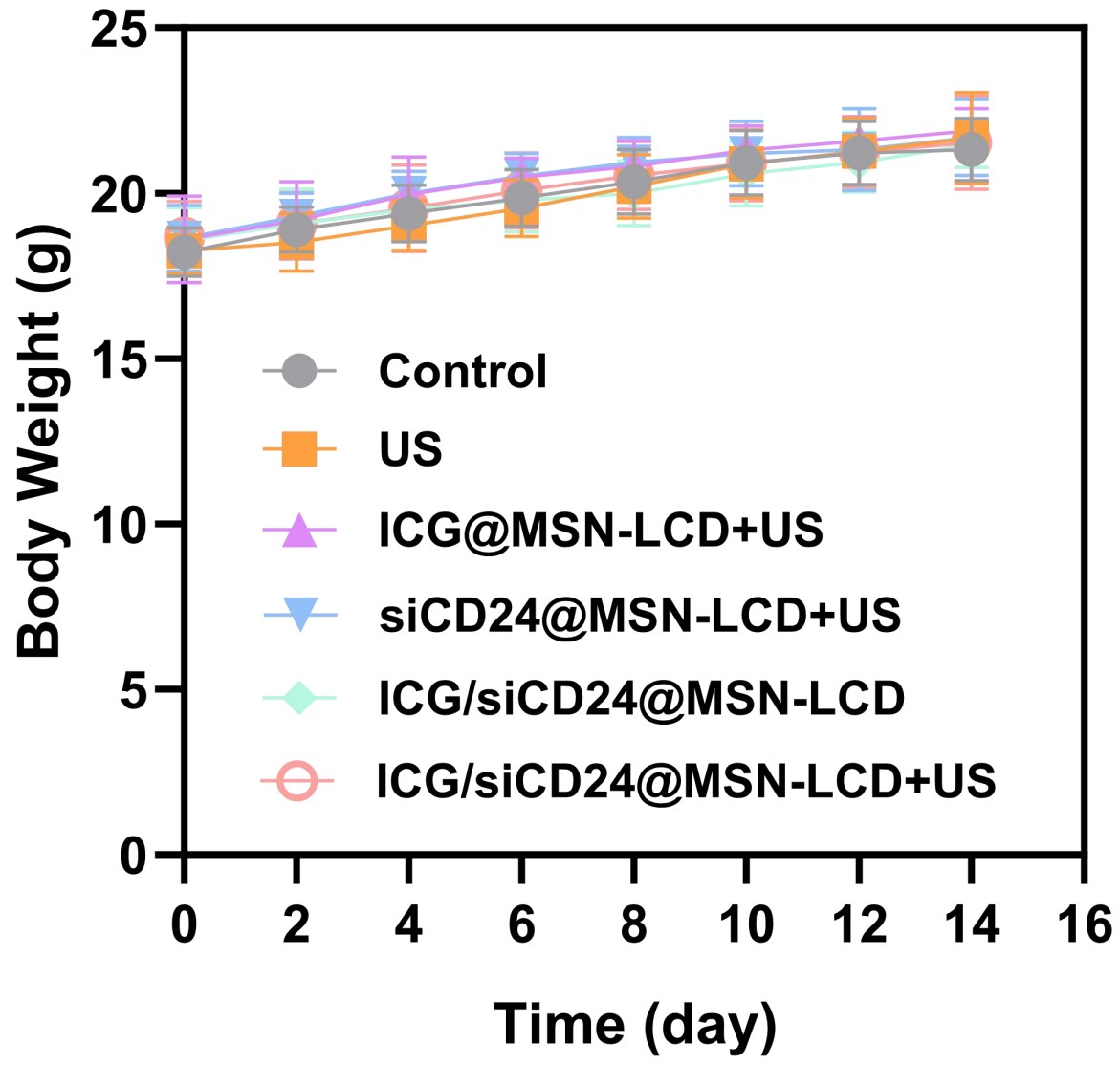

Figure S32. Body weight changes in orthotopic tumor-bearing mice across treatment groups.
